# Networks of habenula-projecting cortical neurons regulate cocaine seeking

**DOI:** 10.1126/sciadv.abj2225

**Published:** 2021-11-05

**Authors:** Victor P. Mathis, Maya Williams, Clementine Fillinger, Paul J. Kenny

**Affiliations:** Nash Family Department of Neuroscience, Icahn School of Medicine at Mount Sinai, New York, NY 10029, USA.

## Abstract

How neurons in the medial prefrontal cortex broadcast stress-relevant information to subcortical brain sites to regulate cocaine relapse remains unclear. The lateral habenula (LHb) serves as a “hub” to filter and propagate stress- and aversion-relevant information in the brain. Here, we show that chemogenetic inhibition of cortical inputs to LHb attenuates relapse-like reinstatement of extinguished cocaine seeking in mice. Using an RNA sequencing–based brain mapping procedure with single-cell resolution, we identify networks of cortical neurons that project to LHb and then preferentially innervate different downstream brain sites, including the ventral tegmental area, median raphe nucleus, and locus coeruleus (LC). By using an intersectional chemogenetics approach, we show that inhibition of cortico-habenular neurons that project to LC, but not to other sites, blocks reinstatement of cocaine seeking. These findings highlight the remarkable complexity of descending cortical inputs to the habenula and identify a cortico-habenulo-hindbrain circuit that regulates cocaine seeking.

## Teaser

Cortical neurons that project to habenula then locus coeruleus regulate cocaine relapse.

## INTRODUCTION

The medial prefrontal cortex (mPFC) plays key roles in decision-making, working memory, and other higher-order executive functions (*1*). The mPFC processes multimodal internal and external stimuli then exerts “top-down” control over midbrain and hindbrain regions to coordinate behavioral output (*2*, *3*). Stress modifies information processing in the mPFC, which triggers behavioral adaptations including alterations in reward seeking (*4*–*7*). Cocaine and other drugs of abuse can profoundly affect mPFC structure and function (*8*, *9*), including how mPFC neurons respond to stressful stimuli (*10*–*13*). This action is thought to contribute to vulnerability to cocaine relapse provoked by stressful events during periods of abstinence (*12*, *13*). However, the circuit-level mechanisms by which mPFC neurons propagate stress-relevant information to subcortical brain sites to influence cocaine relapse remain incompletely understood, in large part because of the complexity of the mPFC “connectome” with downstream brain regions (*14*–*16*).

The lateral habenula (LHb) receives aversion-related information from hypothalamic, limbic, and basal ganglia brain sites (*17*) and encodes negative reward prediction errors that modulate decision-making processes (*18*–*20*), particularly under stressful conditions (*21*–*28*). Chemogenetic silencing of these neurons decreases cocaine relapse–like behavior in rodents (*29*), suggesting that the mPFC and LHb may interact to regulate cocaine seeking. Consistent with this possibility, the LHb receives direct input from the mPFC (*30*–*32*), but little is known about the function of the cortico-habenular circuit (*33*, *34*). The mPFC also sends projections to many of the same midbrain and hindbrain regions involved in reward seeking to which LHb neurons project (*35*–*37*), with mPFC and LHb inputs sometimes converging on the very same neurons in these sites (*35*). Here, we tested the hypothesis that cortico-habenular neurons in the mPFC coordinate the activity of the LHb and the broader system of LHb-regulated circuits in the brain to organize behavioral responses to noxious stimuli and thereby control stress-precipitated cocaine seeking.

## RESULTS

### mPFC_➔LHb_ neurons signal the occurrence of stressful events

**Fig. 1.**
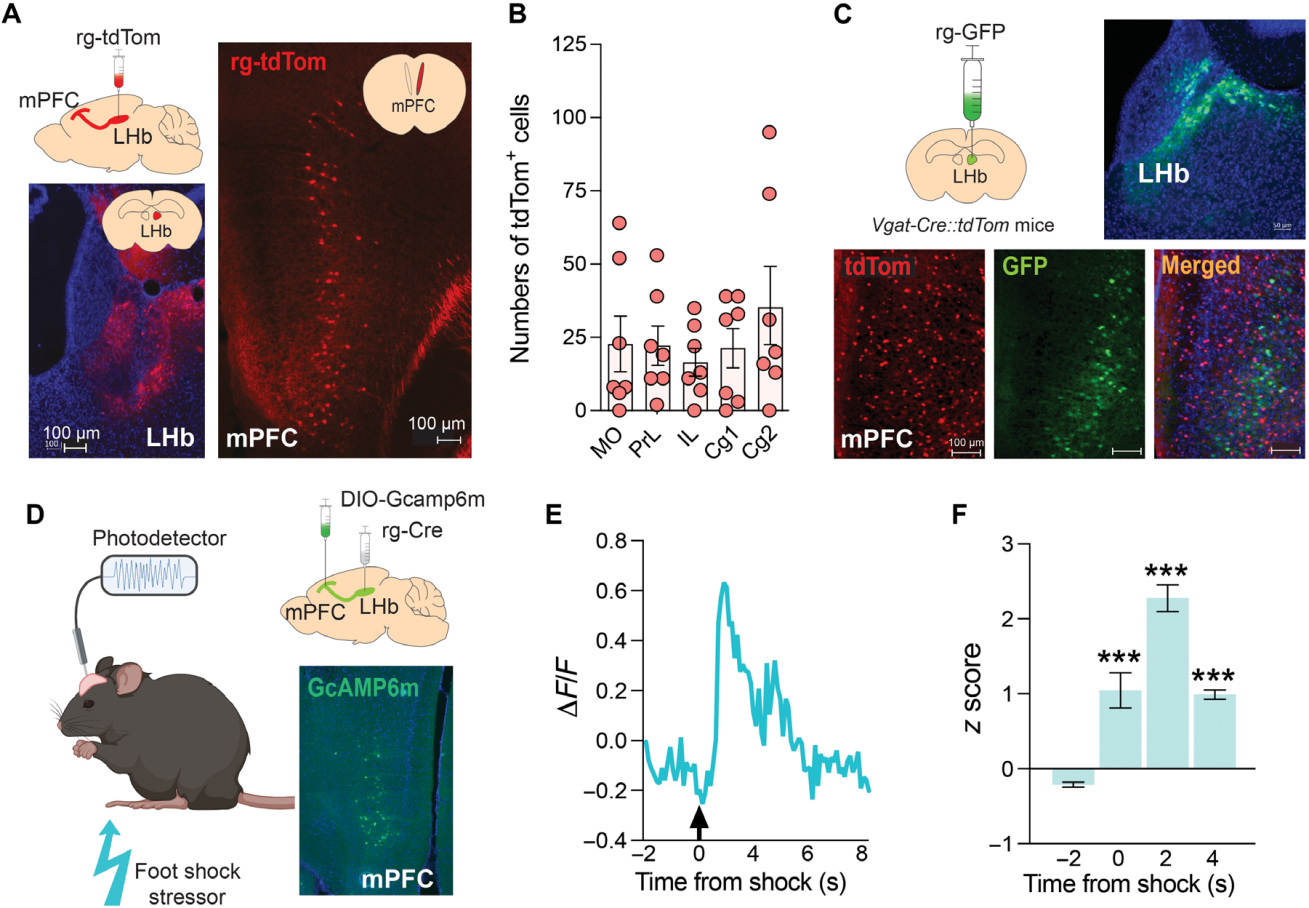
mPFC_➔LHb_ neurons signal the occurrence of stressful events. (**A**) Graphical representation of intracranial injection procedure to label cortico-habenular neurons (top left). Representative fluorescence from tdTom^+^ neurons in the LHb after local injection of rg-tdTom (bottom left). Representative tdTom^+^ cells in the mPFC of mice that received intra-LHb injection of rg-tdTom (right). A total of *n* = 7 mice were imaged. (**B**) Numbers of tdTom^+^ cells detected throughout the mPFC. MO, medio-orbital cortex; PrL, prelimbic cortex; IL, infralimbic cortex; Cg1, anterior cingulate cortex region 1; Cg2, anterior cingulate cortex region 2. (**C**) Graphical representation of intracranial injection procedure to label cortico-habenular neurons in *Vgat-Cre::tdTom* reporter mice (top left). Representative fluorescence from GFP^+^ neurons in the LHb after local injection of rg-GFP (top right). Representative fluorescence image showing that GFP was detected exclusively in non-tdTom^+^ cells in the mPFC of *Vgat-Cre::tdTom* mice injected with rg-GFP in the LHb (bottom). A total of *n* = 4 *Vgat-Cre::tdTom* mice were imaged. (**D**) Schematic of photometry-based in vivo calcium imaging procedure in mice (left). Graphical representation of intracranial injection procedure to express GCaMP6m in cortico-habenular neurons (top right). Representative GCaMP6m expression in the mPFC of mice used in the photometry experiment (bottom right). (**E**) Representative trace showing increase in GCaMP6m-derived fluorescence after delivery of a noxious foot-shock stressor. A total of *n* = 3 mice were recorded. (**F**) *z* score representation of calcium response to foot shock [one-way ANOVA: *F*_(3,80)_ = 44.66, *P* < 0.0001; ***P < 0.001 compared with −2 s time point].

First, to confirm that mPFC neurons project to the LHb, we delivered unilateral injections of a retrogradely traveling AAV to express tdTomato (rg-tdTom) into the LHb of wild-type mice (Fig. 1A). We detected large numbers of rgAAV-tdTom–positive cells (rg-tdTom^+^) throughout the mPFC of these animals (Fig. 1A), particularly in deeper cortical layers known to provide long-range projections to subcortical brain regions (Fig. 1A). rg-tdTom^+^ cells were distributed across the entire rostrocaudal axis of the mPFC, and along the dorsoventral plane (Fig. 1B), suggesting that neurons located in most mPFC compartments send projections to the LHb (mPFC_➔LHb_ neurons). Injection of the retrograde tracer cholera toxin subunit B conjugated to a red fluorescent tag (CTb-598) into the LHb of mice similarly resulted in large numbers of fluorescent cells throughout the mPFC (fig. S1). Injection of a retrograde AAV to express green fluorescent protein (rg-GFP) into the LHb of *Vgat-tdTom* reporter mice, in which GABAergic neurons can be identified by tdTom^+^ fluorescence, resulted in GFP expression exclusively in non-GABAergic (tdTom-negative) cells in the mPFC (Fig. 1C), suggesting that mPFC_➔LHb_ neurons are likely glutamatergic in nature. The mPFC and LHb are known to coordinate behavioral adaptations to noxious and stressful stimuli (*38*, *39*). Therefore, we hypothesized that stress-relevant information may be routed to the LHb in part by mPFC_➔LHb_ neurons. To test this hypothesis, we used fiber photometry to monitor changes in the activity of mPFC_➔LHb_ neurons in response to stressful environmental stimuli (Fig. 1D). We injected a retrograde AAV to express Cre recombinase (rg-Cre) into the LHb of mice, and in the same animals, we injected AAV-DIO-GcAMP6m into the mPFC, which expresses the genetically encoded calcium sensor GCaMP6m in a Cre-dependent manner (Fig. 1D). Delivery of a noxious foot-shock stressor markedly but transiently increased calcium signaling in the mPFC of these mice (Fig. 1, E and F, and fig. S2). These findings are consistent with a prominent projection from the mPFC to the LHb and suggest that these cortico-habenular neurons signal the occurrence of stressful events and can route this information to the LHb.

### mPFC_➔LHb_ neurons regulate cocaine seeking: Intravenous self-administration

**Fig. 2.**
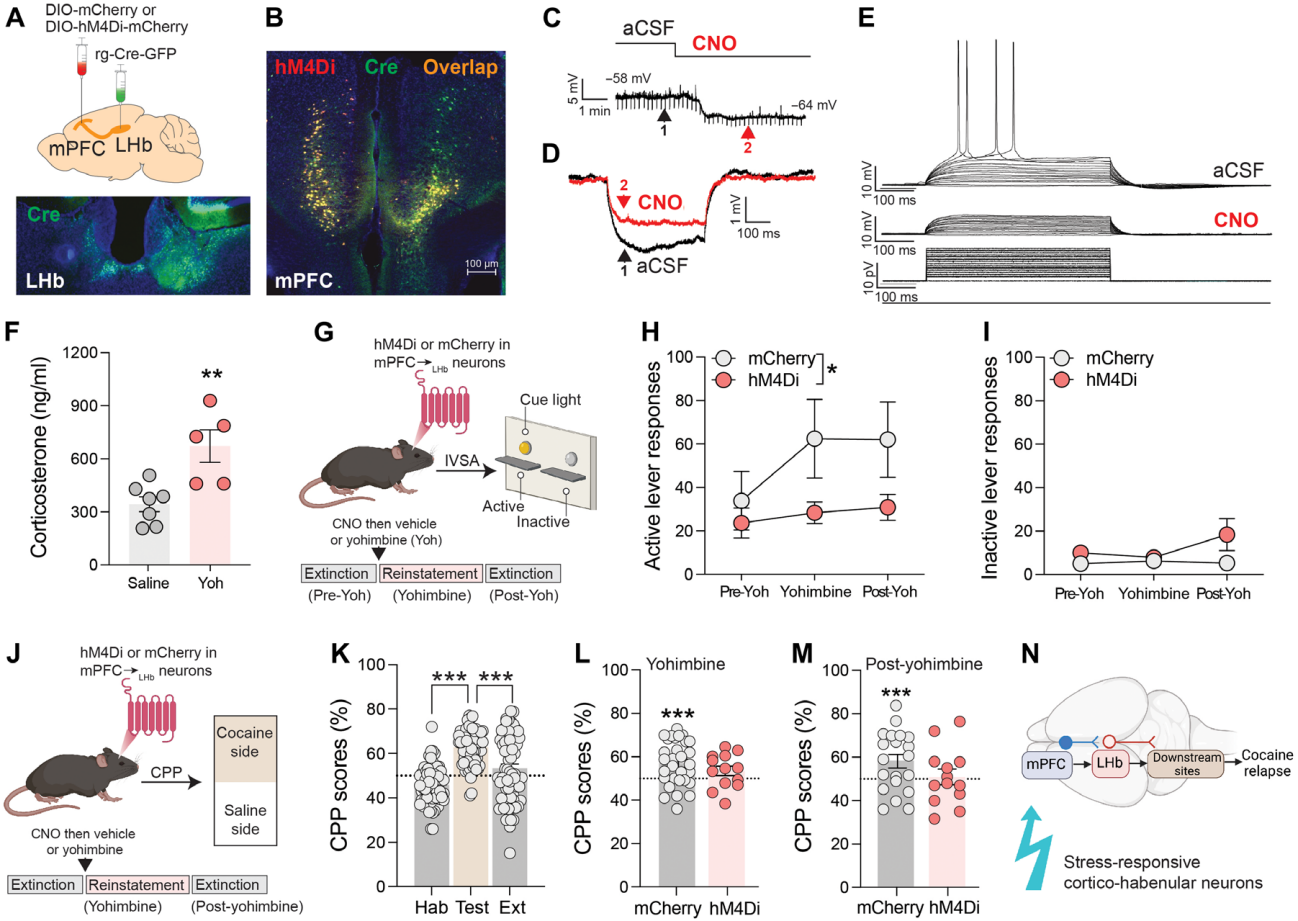
mPFC_➔LHb_ neurons regulate cocaine seeking. (**A**) Summary of virus injection procedure and representative fluorescence image of GFP^+^ neurons in the LHb. (**B**) Representative image from the mPFC of virus-injected mice. (**C**) Time course of CNO-induced hyperpolarization of membrane potential in hM4Di-expressing mPFC neurons. (**D**) Example of CNO-induced reduction of whole-cell membrane resistance. (**E**) Representative traces of voltage changes in whole-cell mode in hM4Di-expressing mPFC neurons depolarized by current injections (500 ms; 10 to 80 pA in 5-pA steps) before [artificial cerebrospinal fluid (aCSF)] and after CNO (20 μM). (**F**) Mean (±SEM) plasma corticosterone levels in yohimbine-treated (Yoh; *n* = 5) and saline-treated (*n* = 7) mice. **P = 0.005, unpaired two-tailed *t* test. (**G**) Summary of intravenous cocaine self-administration (IVSA) procedure. After extinction training, mice were injected with CNO (3 mg kg^−1^) and then yohimbine (2 mg kg^−1^), and responding was recorded. (**H**) Yohimbine reinstated cocaine seeking in mCherry but not hM4Di mice [main effect of *Virus* during yohimbine and post-Yoh sessions; *F*_(1,16)_ = 5.042, *P = 0.0392]. Data are presented as mean (±SEM) responses on active lever. (**I**) Yohimbine did not alter inactive lever responding [*F*_(1,16)_ = 1.573, *P* = 0.2278]. Data are presented as means (±SEM). (**J**) Summary of conditioned place preference (CPP) procedure and experimental design. (**K**) Mean (±SEM) percentage of time spent in cocaine-paired side during habituation (Hab), preference test for cocaine-paired side (Test), and after extinction sessions (Ext) in mice expressing hM4Di (*n* = 11) or mCherry (*n* = 7) in cortico-habenular neurons. ***P < 0.001, unpaired two-tailed *t* test. (**L**) Mean (±SEM) % time spent in cocaine-paired side during reinstatement session in hM4Di (*n* = 11) and mCherry (*n* = 7) mice injected with CNO and then yohimbine. ***P < 0.001, one-sample *t* test. (**M**) Mean (±SEM) % time spent in cocaine-paired side on the day after reinstatement session. ***P < 0.001, one-sample *t* test. (**N**) Graphical representation of cortico-habenular neurons highlighting their responsiveness to stressful stimuli and their involvement in stress-induced reinstatement of cocaine seeking (cocaine relapse).

Next, we investigated the role of mPFC_➔LHb_ neurons in regulating stress-induced reinstatement of cocaine seeking in mice. We infused rg-Cre-GFP into the LHb and AAV-DIO-hM4Di-mCherry (or AAV-DIO-mCherry) into the mPFC of mice (Fig. 2A) to express the inhibitory hM4Di DREADD receptor (or mCherry) specifically in mPFC_➔LHb_ neurons (Fig. 2B). Using patch-clamp recordings, we found that the DREADD receptor agonist clozapine-*N*-oxide (CNO) hyperpolarized mPFC_➔LHb_ neurons and reduced their membrane resistance, which decreased voltage responses to depolarizing current steps (Fig. 2, C to E), confirming that activation of hM4Di reduced the activity of cortico-habenular neurons. Chemogenetic inhibition of mPFC_➔LHb_ neurons did not alter cocaine intake in mice that lever-pressed for intravenous cocaine infusions (0.3 mg kg^−1^ per infusion) under a fixed-ratio 5 time-out 20-s (FR5TO20) schedule of reinforcement (fig. S3). This suggests that mPFC_➔LHb_ neurons are unlikely to regulate the reinforcing properties of cocaine. We then modified the response contingencies such that cocaine infusions and cocaine-paired conditioned stimuli (i.e., cue light activated during infusions) were omitted when mice successfully completed the FR5TO20 schedule on the drug-paired “active” lever, and instead, their lever pressing no longer had any scheduled consequences (similar to the “inactive” lever). This resulted in a gradual extinction of lever-pressing behavior across daily sessions (fig. S3). Chemogenetic inhibition of mPFC_➔LHb_ neurons did not modify responding under extinction conditions (fig. S3), suggesting that mPFC_➔LHb_ neurons are not involved in the learning processes by which cocaine-seeking behaviors are extinguished.

Yohimbine is an α2 adrenoceptor receptor antagonist that precipitates stress-like behavioral and physiological responses (*40*, *41*) and triggers relapse-like cocaine-seeking behavior in rodents and cocaine craving in abstinence of human cocaine users (*42*, *43*). We used yohimbine as a “pharmacological stressor” because other stressors such as foot shock failed to reliably reinstate extinguished cocaine seeking in our mice (fig. S4). We found that a dose of yohimbine (2 mg kg^−1^) that elevated blood corticosterone levels (Fig. 2F) reinstated otherwise extinguished responding on the previously active lever under extinction conditions (Fig. 2, G and H) but did not alter responding on the inactive lever (Fig. 2I). Chemogenetic inhibition of mPFC_➔LHb_ neurons blocked yohimbine-precipitated reinstatement of cocaine-seeking responses on the active lever (Fig. 2H) but did not alter responding on the inactive lever (Fig. 2I).

Recent reports suggest that yohimbine can reinstate drug seeking through stress-independent mechanisms by enhancing the motivational properties of drug-paired conditioned stimuli (*44*, *45*). Therefore, we investigated whether mPFC_➔LHb_ neurons also regulate cocaine seeking precipitated by cocaine-paired conditioned stimuli. However, chemogenetic inhibition of mPFC_➔LHb_ neurons had no effects on cue-induced reinstatement of cocaine seeking (fig. S5). Chemogenetic inhibition of mPFC_➔LHb_ neurons similarly had no effects on the number of food rewards (25 mg) earned by mice responding under the same FR5TO20 schedule of reinforcement (fig. S6). The mPFC and LHb are known to regulate “behavioral flexibility,” in which behavioral output is modified in a dynamic manner in response to changes in the contingencies that deliver rewards or withhold punishers (*46*, *47*). We found that silencing of mPFC_➔LHb_ neurons did not alter the ability of mice to modify their responding for food rewards in a dynamic manner when we switched the active and inactive lever assignments (fig. S6). Hence, nonspecific deficits in behavioral performance or an inability to modify behavior in a goal-directed manner are unlikely to account for the reduced cocaine-seeking behavior in mice after inhibition of mPFC_➔LHb_ neurons. Together, these findings suggest that mPFC_➔LHb_ neurons regulate stress-induced reinstatement of cocaine seeking.

### mPFC_➔LHb_ neurons regulate cocaine seeking: Place conditioning

Next, we sought to confirm a role for mPFC_➔LHb_ neurons in regulating cocaine seeking using a conditioned place preference (CPP) procedure (Fig. 2J). In this procedure, mice were habituated to a two-chamber CPP apparatus and then trained to associate one chamber with the effects of cocaine injections and the other with saline injections (Fig. 2, J and K). On a test day, mice were permitted to freely explore the apparatus, with greater time spent in the cocaine-paired chamber relative to the saline-paired chamber considered a measure of cocaine-seeking behavior (Fig. 2, J and K). We found that chemogenetic inhibition of mPFC_➔LHb_ neurons during the cocaine conditioning sessions did not alter subsequent preference for the cocaine-paired side on the test day (fig. S7). This is consistent with our intravenous cocaine self-administration data (see fig. S3) and suggests that mPFC_➔LHb_ neurons are unlikely to regulate the rewarding properties of cocaine. Next, we trained a new cohort of mice in the cocaine CPP procedure and then extinguished their preference for the cocaine-paired side by allowing them to freely explore the CPP apparatus in a drug-free state across daily sessions. In mCherry-expressing control mice, yohimbine reinstated the otherwise extinguished preference for the cocaine-paired side (Fig. 2L). In hM4Di-expressing mice, chemogenetic silencing of mPFC_➔LHb_ neurons prevented yohimbine-induced reinstatement of preference for the cocaine-paired side (Fig. 2L and fig. S8). Preference for the cocaine-paired side persisted in the yohimbine-treated control mice for at least 24 hours, an effect also absent in the yohimbine-treated hM4Di mice (Fig. 2M).

Reinstatement of preference for the cocaine-paired side of the CPP apparatus was not observed in cocaine-conditioned control mice injected with saline instead of yohimbine on the test day (fig. S9). Mice injected with saline instead of cocaine during the conditioning sessions did not demonstrate any change in preference for either side of the CPP apparatus on the test day when injected with saline or yohimbine (fig. S9). Similarly, control mice injected with yohimbine instead of cocaine during the conditioning sessions did not demonstrate a preference for either side of the CPP apparatus on the test day (fig. S9). These data suggest that the reinstatement of preference for the cocaine-paired side of the CPP apparatus reflected relapse-like cocaine-seeking behavior.

Spatial memory deficits and increases in anxiety can disrupt cocaine-induced place conditioning (*48*, *49*). The mPFC (*50*) and LHb (*51*) regulate spatial learning and anxiety-related behaviors. However, chemogenetic inhibition of mPFC_➔LHb_ neurons during the acquisition of new fear memories did not alter freezing behavior during the conditioning session, or the later expression of context- or cue-conditioned freezing behavior, in a classical fear conditioning procedure (fig. S10). In a separate group of mice, we found that chemogenetic inhibition of mPFC_➔LHb_ neurons during the postconditioning test sessions did not alter context- or cue-induced freezing behavior (fig. S11). Inhibition of mPFC_➔LHb_ neurons did not alter anxiety-like behavior or locomotor activity in mice tested in a light-dark box procedure (fig. S12). Similarly, inhibition of mPFC_➔LHb_ neurons did not alter the total distance traveled or resting time in an open-field apparatus (fig. S13). This suggests that reduced cocaine seeking in the CPP procedure after silencing of mPFC_➔LHb_ neurons is unlikely to reflect nonspecific abnormalities in cognitive, emotional, or locomotor behaviors. Overall, these findings support a key role for mPFC_➔LHb_ neurons in regulating relapse-like cocaine-seeking behaviors (Fig. 2N).

### Defining the connectomes of individual mPFC_➔LHb_ neurons

**Fig. 3.**
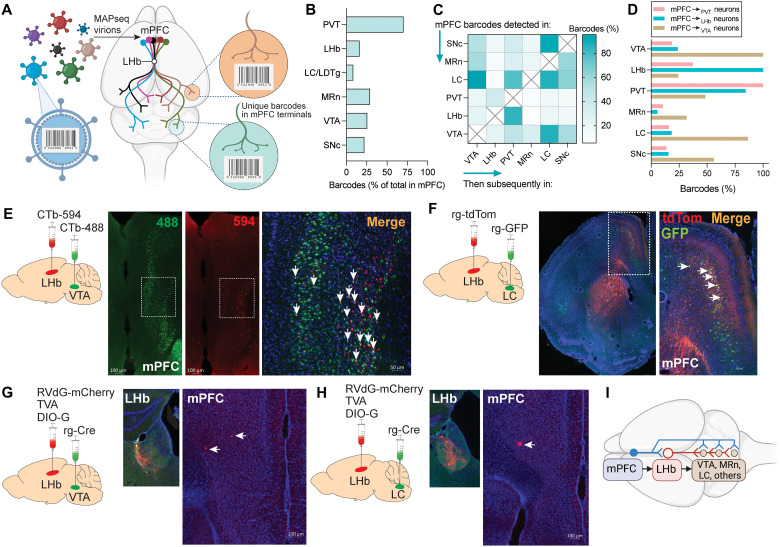
Connectomes of mPFC_➔LHb_ neurons. (**A**) Summary of MAPseq procedure. (**B**) Percentage of barcodes detected in the mPFC also detected in brain sites listed on *y* axis. Data were collected from *n* = 2 mice. (**C**) Heatmap representation of mPFC barcodes (% total number) detected in brain regions shown on *y* axis and in regions shown on *x* axis. (**D**) Histogram of connectome of mPFC_➔PVT_, mPFC_➔VTA_, and mPFC_➔LHb_ neurons. Shown is the distribution of barcodes (%) for mPFC cells that project to the PVT, VTA, and LHb. (**E**) Summary of injection procedure to label cortical-habenular neurons that project to VTA and fluorescence image of mPFC from mice injected with CTb retrograde tracers in the LHb and VTA. Shown are CTb-594 (red; mPFC_➔LHb_ neurons)–labeled and CTb-488 (green; mPFC_➔VTA_ neurons)–labeled cells. White arrows identify dual-labeled cells (yellow; mPFC_➔LHb_^VTA^ neurons) in the mPFC. A total of *n* = 5 mice were imaged. (**F**) Summary of injection procedure to label cortical-habenular neurons that project to the LC and associated fluorescence image of mPFC from injected mice (red; mPFC_➔LHb_ neurons) and GFP (green; mPFC_➔VTA_ neurons). White arrows identify dual-labeled cells (yellow; mPFC_➔LHb_^LC^ neurons) in the mPFC. A total of *n* = 5 mice were imaged. (**G**) Injection procedure (the tracing the relationship between input and output method, also known as the TRIO method) to label cortico-habenular neurons that project to VTA. Shown are mCherry^+^ cells in the mPFC, which are cortico-habenular neurons that synapse into LHb neurons that project to VTA. A total of *n* = 4 mice were imaged. (**H**) Injection procedure to label cortico-habenular neurons that project to the LC. mCherry^+^ cells were detected in the mPFC (right), which are cortico-habenular neurons that synapse into LHb neurons that project to the LC. A total of *n* = 3 mice were imaged. (**I**) Graphical representation of the cortico-habenular connectome. A population of mPFC neurons (shown in blue) projects concurrently to the LHb and then to the same monoaminergic brain centers to which LHb neurons also project (shown in red).

mPFC and LHb neurons send prominent inputs to brain sites involved in stress adaptation, aversion, and reward seeking, most notably monoaminergic centers in midbrain and hindbrain (*37*, *52*, *53*). In some cases, mPFC and LHb projections converge onto the same individual neurons in these sites (*35*). Hence, cortico-habenular neurons are positioned to exert simultaneous indirect and direct control over LHb-regulated monoaminergic centers in the brain via projections to the LHb or the downstream sites to which LHb neurons project, respectively. Therefore, we hypothesized that mPFC_➔LHb_ neurons regulate cocaine seeking by providing “higher-order” cortical inputs to LHb-regulated brain stress and aversion systems. As a first step to testing this hypothesis, we profiled the connectomes of mPFC_➔LHb_ neurons with single-cell resolution using the unbiased multiplexed analysis of projections by sequencing (MAPseq) technique (Fig. 3A) (*54*). MAPseq is an RNA sequencing–based technology in which neurons are infected with a neurotropic and propagation-incompetent Sindbis virus to express GFP (to confirm infection in targeted brain site) and a unique genetic barcode [random RNA sequence 12 nucleotides (nt) in length], with each barcode expressed in only a single neuron (Fig. 3A) (*15*, *54*). Single-neuron expression is achieved by virtue of the enormous diversity of MAPseq barcodes (~17 million unique identifiers) (Fig. 3A) (*55*), which far exceeds the total number of neurons in the small volume of cortex into which the MAPseq virus is infused (~1 mm^3^ volume; ~90,000 neurons) (Fig. 3A) (*56*). It has been shown that >99% of neurons in a targeted brain region are uniquely labeled by the MAPseq virus (*54*). Detection of a barcode in both the site of MAPseq virus injection and in other brain sites suggests that the barcoded neuron projects to those other sites (Fig. 3A) (*15*, *54*).

Using MAPseq, we profiled the connectivity of mPFC neurons that project to the LHb and then to monoaminergic centers in the midbrain and hindbrain, including the ventral tegmental area (VTA), substantia nigra pars compacta (SNc), median raphe nucleus (MRn), and locus coeruleus (LC) (which included some tissue from the adjacent laterodorsal tegmental nucleus). A total of 141 unique barcodes were detected in the mPFC and in one or more of the downstream brain regions that we sequenced. Among all barcoded cells in the mPFC, a large proportion (70%) projected to the paraventricular nucleus of the thalamus (mPFC_➔PVT_ neurons) (Fig. 3B). This is consistent with the known projection profiles of mPFC neurons in deeper cortical layers, almost all of which project to various thalamic nuclei (*57*). Of these mPFC_➔PVT_ neurons, ~37% also coprojected to the LHb (Fig. 3, C and D). Relatively high proportions of mPFC neurons projected to the VTA (26%) or MRn (29%), whereas a lower proportion projected to the LC (9%) (Fig. 3B). A moderate proportion of mPFC neurons projected to the LHb (16%) (mPFC_➔LHb_ neurons) (Fig. 3B). A very large fraction of the barcodes detected in cortico-habenular neurons were also detected in the PVT (84%) (Fig. 3, C and D), suggesting that mPFC_➔LHb_ neurons may represent a subpopulation of a much larger group of mPFC_➔PVT_ neurons. A sizeable proportion of mPFC_➔LHb_ neurons coprojected to the VTA (24%; mPFC_➔LHb_^VTA^ neurons) or LC (18%; mPFC_➔LHb_^LC^ neurons), but a relatively low proportion coprojected to the MRn (6%; mPFC_➔LHb_^MRn^ neurons) (Fig. 3, C and D). This projection profile of mPFC_➔LHb_ neurons was similar to that of mPFC_➔PVT_ neurons (Fig. 3, C and D) but differed markedly from that of mPFC neurons that projected to the VTA (mPFC_➔VTA_ neurons). Compared with mPFC_➔LHb_ neurons, mPFC_➔VTA_ neurons had lower connectivity with PVT (48%) and much higher connectivity with LC (86%) and MRn (~32%) (Fig. 3, C and D). These findings suggest that populations of mPFC neurons project concurrently to the LHb and to the same monoaminergic brain centers to which LHb neurons also project. This wiring plan positions cortico-habenular neurons to provide top-down direct and indirect executive control over habenular-regulated brain stress and aversion circuits.

We sought to confirm the “multiplexed” nature of cortico-habenular connectomes by using a combination of retrograde tracers. First, we infused CTb-488 into the VTA and CTb-594 into the LHb of the same mice. We detected CTb-594^+^ cells (mPFC_➔LHb_ neurons) primarily in deeper layers of the mPFC and CTb-488^+^ cells (mPFC_➔VTA_ neurons) primarily in superficial layers (Fig. 3E). However, double-labeled neurons were detected, most of which were located in deeper cortical layers (Fig. 3E). We replicated this pattern of expression when rg-tdTom was infused into the LHb and rg-GFP into the VTA of the same mice (fig. S14). Similarly, dual tdTom^+^/GFP^+^ cells were detected in the mPFC of mice injected with rg-tdTom into the LHb and with rg-GFP into the LC (Fig. 3F).

Next, we investigated whether the same LHb neurons that receive synaptic input from mPFC_➔LHb_ neurons also send efferent input to the monoaminergic centers to which mPFC_➔LHb_ neurons project. In this manner, mPFC_➔LHb_ neurons can provide direct and indirect input to habenular-regulated brain systems. For this purpose, we used the glycoprotein-deleted rabies virus (RVdG) system for monosynaptic tracing (*58*, *59*). We focused on the VTA first because this site received more substantial input from mPFC_➔LHb_ neurons than the other downstream monoaminergic centers we examined (Fig. 3, C and D). First, we injected rg-Cre into the VTA of mice (Fig. 3G). We also injected an AAV to express the TVA receptor (TVA) and an AAV to express rabies glycoprotein in a Cre-dependent manner (DIO-G) into the LHb of the same mice (Fig. 3G). Then, 3 weeks later, we injected an AAV into the LHb of the mice to express RVdG-mCherry (Fig. 3G). According to this configuration, only LHb neurons that project to the VTA (LHb_➔VTA_ neurons) can express Cre recombinase and thereby generate functional rabies virus particles to label any cortical neurons that provide direct synaptic inputs onto these neurons. We detected mCherry^+^ cells in the mPFC of these mice (Fig. 3G), confirming that mPFC_➔LHb_ neurons synapse directly onto LHb_➔VTA_ neurons. When we injected rg-Cre into the MRn instead of VTA of a new group of mice, with the other components of the injection configuration unchanged, we again detected mCherry^+^ cells in the mPFC (Fig. 3H). This confirms that mPFC_➔LHb_ neurons also synapse directly onto LHb_➔LC_ neurons. Hence, groups of mPFC neurons provide concurrent synaptic input to neurons in the LHb and the neurons in downstream monoaminergic brain centers to which these same LHb neurons project. Together, these findings reveal the complex wiring architectures of cortico-habenular neurons, in which individual mPFC neurons provide multiplexed inputs to LHb neurons and LHb-regulated neurons located in stress- and aversion-relevant centers in the midbrain and hindbrain (Fig. 3I).

### mPFC_➔LHb_^LC^ neurons regulate cocaine-seeking behavior

**Fig. 4.**
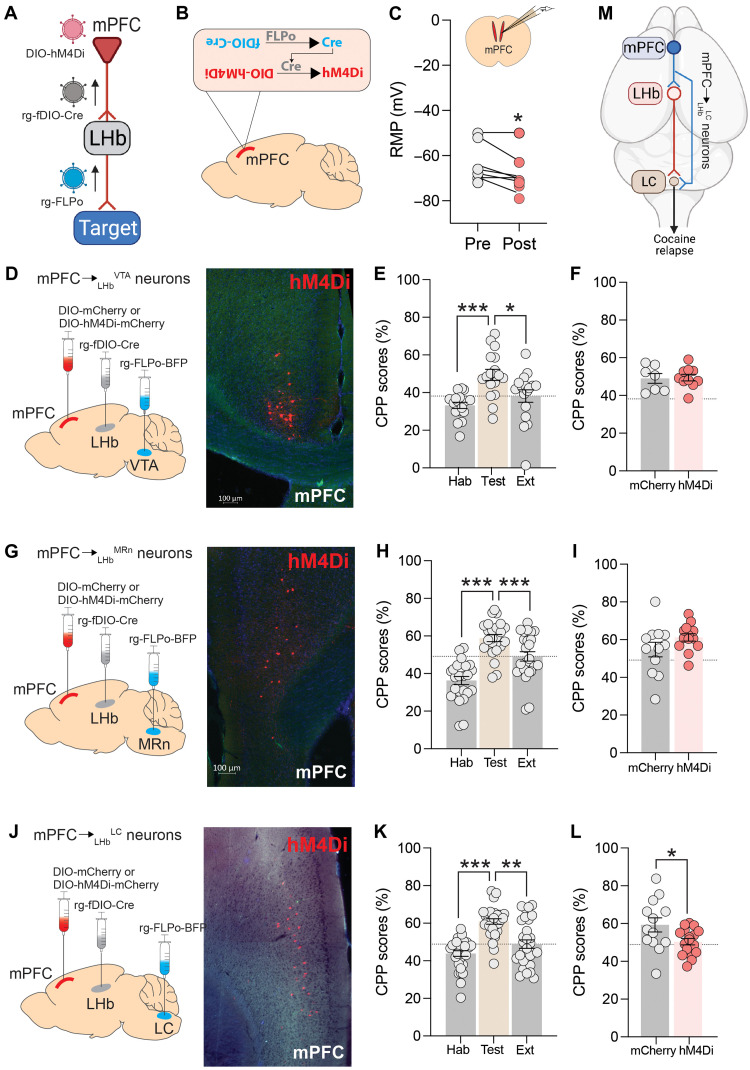
mPFC_➔LHb_^LC^ neurons regulate cocaine seeking. (**A**) Summary of intersectional genetic approach to express hM4Di in cortico-habenular neurons that project to the VTA, MRn, or LC. (**B**) Only cortico-habenular neurons that project to targeted downstream sites can contain FLPo and fDIO-Cre necessary to express DIO-hM4Di. (**C**) CNO hyperpolarized mPFC neurons that express hM4Di. *P < 0.05, paired *t* test. RMP, resting membrane potential. (**D**) Summary of procedure to express hM4Di-mCherry (or only Cherry) in mPFC_➔LHb_^VTA^ neurons. Representative image of hM4Di-mCherry^+^ cells in mPFC_➔LHb_^VTA^ neurons. (**E**) Mean (±SEM) time (%) spent in the cocaine-paired side of CPP apparatus during habituation, testing, and extinction sessions in mice expressing hM4Di (*n* = 10) or mCherry (*n* = 7) in mPFC_➔LHb_^VTA^ neurons. ***P < 0.001 and *P < 0.05, unpaired two-tailed *t* tests. (**F**) Mean (±SEM) time (%) spent in cocaine-paired side during reinstatement in hM4Di (*n* = 11) and mCherry (*n* = 7) mice injected with CNO and then yohimbine. (**G**) Summary of strategy to target mPFC_➔LHb_^MRn^ neurons and fluorescence image of hM4Di-mCherry in mPFC_➔LHb_^MRn^ neurons. (**H**) Mean (±SEM) time (%) spent in cocaine-paired side during habituation, testing, and extinction sessions in hM4Di (*n* = 13) and mCherry (*n* = 12) mice. ***P < 0.001, unpaired two-tailed *t* tests. (**I**) Mean (±SEM) time (%) spent in cocaine-paired side during reinstatement session in hM4Di (*n* = 13) and mCherry (*n* = 12) mice injected with CNO and then yohimbine. (**J**) Summary of strategy to target mPFC_➔LHb_^LC^ neurons and representative image of hM4Di-mCherry in mPFC_➔LHb_^LC^ neurons. (**K**) Mean (±SEM) time (%) spent in cocaine-paired side during habituation, testing, and extinction sessions in hM4Di (*n* = 17) and mCherry (*n* = 13) mice. ***P < 0.001, unpaired two-tailed *t* tests. **P < 0.01.(**L**) Mean (±SEM) time (%) spent in cocaine-paired side during reinstatement session in hM4Di (*n* = 17) and mCherry (*n* = 13) mice injected with CNO and then yohimbine. *P < 0.05, unpaired two-tailed *t* tests. (**M**) Graphical representation of the cortico-habenular neurons that project to the LC and regulate cocaine seeking.

Last, we investigated whether any of the cortico-habenular microcircuits identified above participate in stress-induced cocaine-seeking behavior. To accomplish this, we developed a novel intersectional chemogenetic approach in which we infused rgAAV to express codon-optimized flippase (FLPo) and blue fluorescent protein (rg-FLPo-BFP) into the VTA, MRn, or LC of mice (Fig. 4A). In the same animals, we injected rgAAV to express Cre in an FLPo-dependent manner (rg-dDIO-Cre) into the LHb (Fig. 4A). We also injected a control AAV (DIO-mCherry) or an AAV to express hM4Di in a Cre-dependent manner (DIO-hM4Di-mCherry) into the mPFC of the same mice (Fig. 4A). By configuring our injections in this manner, only those mPFC neurons that provide concurrent monosynaptic input to neurons in the LHb and the VTA, MRn, or LC will express Cre in an FLPo-dependent manner, which can drive Cre-dependent hM4Di (or mCherry) expression in these cells (Fig. 4B). We detected mCherry^+^ cells in the mPFC of DIO-mCherry–treated and DIO-hM4Di-mCherry–treated mice (Fig. 4, D, G, and J) but not in control mice in which we omitted any one of the viruses required for this dual recombinase strategy (fig. S15). This confirmed that the dual recombination procedure successfully expressed hM4Di-mCherry in cortico-habenular neurons that innervate the VTA, MRn, or LC. Using patch-clamp recordings, we confirmed that CNO hyperpolarized these hM4Di-mCherry^+^ mPFC cells (Fig. 4C). Next, we investigated the effects of silencing mPFC_➔LHb_ neurons that project to the VTA, MRn, or LC on cocaine-seeking behavior. Specifically, we conditioned mice to prefer the cocaine-paired side of a two-chamber CPP apparatus, extinguished this preference across daily extinction sessions, and then reinstated preference for the cocaine-paired side by injecting the mice with yohimbine (2 mg kg^−1^). Silencing mPFC_➔LHb_ neurons that project to the VTA (Fig. 4, D to F) or MRn (Fig. 4, G to I) had no effects on reinstatement of cocaine seeking. However, silencing mPFC_➔LHb_ neurons that project to the LC decreased cocaine seeking (Fig. 4, J to L). To confirm the behavioral specificity of this effect, we examined the consequences of silencing mPFC_➔LHb_^VTA^, mPFC_➔LHb_^MRn^, and mPFC_➔LHb_^LC^ neurons on exploratory behavior in an open field. We observed no effects on the total distance traveled or any other behavior measured in the open-field apparatus in these mice (fig. S16). Behavior in a light-dark box test of anxiety was similarly unaffected (fig. S16). This suggests that nonspecific deficits in locomotor activity or anxiety-related behavior are unlikely to account for the decreased cocaine seeking observed when mPFC_➔LHb_^LC^ neurons were inhibited.

As noted above, mPFC neurons regulate contextual learning, deficits in which can disrupt cocaine-induced place conditioning. Silencing mPFC_➔LHb_ neurons, independent of their downstream projections, had no effects on context-induced freezing in mice tested in a fear conditioning procedure (see figs. S11 and S12). As expected, silencing mPFC_➔LHb_^LC^ neurons also had no effects on context-induced freezing (fig. S17). This suggests that cognitive deficits are unlikely to explain the reduced cocaine seeking observed when these cells were chemogenetically inhibited. Silencing mPFC_➔LHb_^VTA^ neurons similarly had no effects on context-induced freezing (fig. S17). Unexpectedly, silencing mPFC_➔LHb_^MRn^ neurons decreased context-induced freezing (fig. S17). The mPFC and MRn are known to regulate contextual learning (*50*, *60*), and emerging evidence suggests that the LHb may also participate (*51*). Hence, these data suggest that the mPFC_➔LHb_^MRn^ neurons contribute to freezing behavior in fear-evoking environment. Overall, these findings identify an important role for mPFC_➔LHb_^LC^ neurons in relapse-like cocaine-seeking behavior and demonstrate the high degree to which subpopulations of cortico-habenular neurons, defined by the downstream sites to which they project, are functionally specialized to regulate discrete and dissociable stress- and aversion-related behaviors (Fig. 4M).

## DISCUSSION

This study reveals the remarkable complexity of cortico-habenular networks in the brain and identifies their important roles in stress-evoked cocaine seeking and other aversion-related behaviors. We found that mPFC neurons that project to the LHb regulate the resumption of otherwise extinguished cocaine seeking in mice, as measured using the intravenous cocaine self-administration and cocaine-conditioned place preference procedures. By mapping the connectomes of individual cortico-habenular neurons, we show that these cells can be parsed into functional subpopulations based on their connectivity with downstream monoaminergic centers in the brain. Notably, only the subpopulation that projects to the LC, but not those that project to the median raphe or VTA, regulates cocaine seeking. By contrast, those cortico-habenular neurons that project to the raphe but not LC or VTA regulate freezing behavior in a threatening environment. These data identify a novel cortico-habenulo-hindbrain circuit involved in relapse-like cocaine seeking. More broadly, our findings suggest that cortical neurons that project to a given brain site, often considered as unitary cortical circuits, can instead have notably different roles in coordinating stress- and addiction-relevant behaviors depending on their multiplexed connectivity with other brain regions, which presumably reflects their specialized contributions to discrete behavioral and physiological processes.

The mPFC is thought to play a prominent role in relapse to cocaine use during periods of abstinence (*61*), particularly in response to stressful events or stimuli. The mPFC coordinates behavioral and physiological responses to stress (*5*, *62*, *63*), and inhibition of mPFC neurons prevents stress-induced reinstatement of cocaine seeking in rats (*64*, *65*). Consumption of cocaine or other addictive drugs can alter mPFC-dependent executive functions, which may increase vulnerability to stress-induced relapse to cocaine use (*66*–*68*). The LHb also responds to stressful and aversive stimuli and is thought to influence mPFC-regulated higher-order cognitive processes in a stress-dependent manner (*18*, *69*, *70*). Moreover, inhibition of LHb neurons decreases cocaine relapse–like behavior in rats (*29*). These observations suggest that interplay between the mPFC and LHb may regulate vulnerability to stress-induced relapse to cocaine seeking. Nevertheless, little is currently known about mPFC-LHb interactions during periods of stress or their potential involvement in drug-seeking behavior. Recent reports suggest that cortico-habenular neurons route context-relevant information to midbrain and hindbrain sites to modulate working memory and social behaviors during times of stress (*33*, *34*). Using in vivo calcium imaging, we found that cortico-habenular neurons signal the occurrence of a noxious/stressful event. This action is similar to LHb neurons, which also show increased activity in response to unanticipated noxious events (*33*). This suggests that cortical neurons are likely to influence habenular responses to stress. Consistent with this possibility, we found that chemogenetic silencing of cortico-habenular neurons blocked yohimbine-precipitated resumption of otherwise extinguished cocaine-seeking behaviors in mice tested in the intravenous self-administration and place-conditioning procedures. By contrast, silencing cortico-habenular neurons had no effects on intravenous cocaine self-administration behavior when the drug was available for consumption or on the development of a cocaine-conditioned place preference. Silencing cortico-habenular neurons also had no effects on cocaine seeking precipitated by a cocaine-paired conditioned stimulus. Hence, cortico-habenular neurons are unlikely to regulate the rewarding and reinforcing actions of cocaine per se but, instead, are specifically involved in cocaine seeking precipitated by stress. We used yohimbine as a pharmacological stressor in our experiments because other stressors such as foot shock or restraint stress failed to reliably reinstate extinguished cocaine seeking in our mice. Yohimbine is an α2 adrenoceptor antagonist that increases sympathetic nervous system transmission and elevates levels of circulating stress hormones including cortisol in humans and corticosterone in rodents (*40*, *41*, *43*, *71*). Moreover, yohimbine reliably reinstates extinguished drug seeking in rodents and triggers craving across different drug classes in humans (*42*, *43*, *72*, *73*). The dose of yohimbine that we used elevated corticosterone levels in our mice, consistent with the induction of a physiological stress response. However, recent reports suggest that yohimbine can precipitate drug seeking independent of its stress-related actions by enhancing the salience of drug-paired conditioned stimuli (*44*, *45*). We found that silencing cortico-habenular neurons attenuated yohimbine-precipitated cocaine seeking in the self-administration procedure (i.e., responding on the previously cocaine-paired lever) even when drug-paired conditioned stimuli were omitted. Silencing cortico-habenular neurons also attenuated cocaine seeking in a CPP procedure in which the relationship between an operant cocaine-seeking response (lever pressing) and delivery of cocaine-paired conditioned stimuli (cue light) was not established. Hence, a parsimonious interpretation of our data is that cortico-habenular neurons regulate cocaine seeking triggered by stress. This interpretation is strengthened by the finding that only those cortico-habenular neurons that project to the LC, a major noradrenergic brain site involved in coordinating behavioral responses to stressful and arousing stimuli (*74*), regulated cocaine seeking in our mice (see below). Nevertheless, it is possible that cortico-habenular neurons participate in some other nonstress behavioral or physiological action of yohimbine that drives relapse-like cocaine-seeking behaviors.

We used MAPseq technology to express RNA barcodes in individual mPFC neurons and high-throughput RNA sequencing to map the connectomes of cortico-habenular neurons with single-cell resolution. We confirmed our MAPseq findings using traditional and rabies-based monosynaptic neuronal tracing techniques. Using these approaches, three major populations of cortico-habenular neurons were discerned: a relatively abundant population that projected to the VTA, a less-abundant population that project to the LC, and a sparse population that projected to the MRn. It is likely that other unique subpopulations of cortico-habenular neurons can be identified on the basis of their projections to stress-relevant brain sites not included in our MAPseq experiments. For example, the LHb projects to the rostromedial tegmental nucleus, lateral hypothalamus, and parabrachial nucleus (*52*, *75*), aversion-related sites to which mPFC neurons also project. Hence, it is possible that subpopulations of cortico-habenular neurons separate from those identified above project to these sites and thereby regulate discrete stress-related behaviors. As noted above, chemogenetic silencing of cortico-habenular neurons in an unbiased manner (i.e., independent of their downstream projections) blocked cocaine-seeking behavior in mice. This action was recapitulated when we used an intersectional chemogenetic approach to specifically silence only those cortico-habenular neurons that project to the LC. By contrast, silencing cortico-habenular neurons that project to the VTA or MRn had no effects on cocaine seeking. Context-induced freezing in a fear conditioning procedure was unaltered in mice when we silenced cortico-habenular neurons in an unbiased manner or silenced only the LC-projecting population. Operant performance, locomotor activity, and anxiety-related behaviors were also unaltered by silencing cortico-habenular neurons. This suggests that nonspecific abnormalities in behavioral or cognitive performance are unlikely to account for the reduced cocaine seeking when mPFC_➔LHb_ or mPFC_➔LHb_^LC^ neurons were inhibited. It is notable that only ~16% of barcoded mPFC neurons projected to the LHb, and only ~18% of these cortico-habenular cells projected to the LC. This suggests that mPFC_➔LHb_^LC^ neurons represent a very small fraction of the total number of cells in the mPFC. It may seem unexpected that such a sparse population of cortical neurons can exert such robust control over cocaine-seeking behaviors. However, this is consistent with single-cell and single-nucleus RNA sequencing studies that are revealing rare and low-abundance cell types in the brain that are functionally important (*76*, *77*). As these RNA sequencing methodologies continue to evolve and are used in conjunction with MAPseq, spatial transcriptomics, and other related technologies, it is likely that large groups of neurons that appear functionally and anatomically homogenous will be parsed into smaller populations of specialized units based on their transcriptomic and connectomic profiles. We anticipate that even the populations of cortico-habenular neurons that project to the VTA, LC, or MRn are likely heterogenous groups related by functionally distinct cells that can be further parsed on the basis of the other brains sites to which they project and the behaviors they control. Hence, the fact that a relatively sparse population of cortico-habenular neurons that project to the LC can exert such robust control over cocaine seeking may reflect a more generalized organizational principle of the mPFC. Specifically, larger groups of seemingly similar cells may instead reflect clusters of highly specialized smaller units that coordinate discrete behavioral responses to stress and other noxious stimuli based on the precise array of their wiring with downstream “effector” brain sites.

Related to the above point, it is interesting that a deficit in context-induced freezing in a fear conditioning procedure was observed only when the sparse population of cortico-habenular neurons that project to the MRn was silenced. By contrast, silencing mPFC_➔LHb_ neurons independent of their downstream projections, or inhibition of those cortico-habenular neurons that project to the LC or VTA, had no effects on context-induced freezing. This suggests that mPFC_➔LHb_^MRn^ neurons are an important subpopulation of cortical cells that participate in context-induced freezing. These data also suggest that discrete populations of cortico-habenular neurons may exert functionally antagonistic effects on contextual-induced freezing, which would explain why unbiased inhibition of mPFC_➔LHb_ neurons had no effects on freezing behavior. Essentially, the contributions of functionally antagonistic populations of cortico-habenular neurons may be masked when the entire circuit is manipulated in a manner independent of the broader connectome of the individual populations of cells. A similar masking of the contributions of functionally antagonistic populations of neurons can be observed when medium spiny neurons (MSNs) in striatum are inhibited indiscriminately, whereas selective inhibition of striatonigral or striatopallidal MSN subpopulations can reveal profound but opposite effects on behavior (*78*). It will be interesting to determine whether other as-yet unidentified populations of cortico-habenular neurons that project to downstream brain sites not included in our experiments also participate in context-induced freezing behavior but in a manner opposite to mPFC_➔LHb_^MRn^ neurons.

In summary, our findings reveal a complex network of connectivity between mPFC neurons that project to the habenula and then to monoaminergic brain sites known to regulate reward-seeking behaviors and adaptations to stress. Cortico-habenular neurons can be parsed into discrete functional subcircuits based on the precise midbrain and hindbrain sites to which they project. Those cortico-habenular neurons that project to the LC, but not those that project to the MRn or VTA, regulate stress-induced reinstatement of cocaine seeking. By contrast, cortico-habenular neurons that project to the MRn, but not those that project to the LC or VTA, regulate behavioral responses to environmental contexts associated with noxious stimuli. We postulate that many discrete populations of cortico-habenular neurons, defined by their projections to different downstream effector sites, play distinct and dissociable roles in coordinating behavioral responses to different classes of stressful and aversive stimuli. One of these populations, which projects to the LC, establishes a previously uncharacterized cortico-habenulo-hindbrain circuit that regulates vulnerability to stress-induced relapse to cocaine-seeking behavior.

## MATERIALS AND METHODS

### Animals and surgeries

Male C57Bl/6J wild-type mice (Jackson) aged 8 to 12 weeks at the start of each experiment were used in accordance with protocols approved by the Institutional Animal Care and Use Committee of the Icahn School of Medicine at Mount Sinai. *Vgat-Cre^+/−^::tdTomato^+/−^* mice were generated by crossing *Vgat-Ires-Cre* mice (stock no. 028862, Jackson Laboratories) with B6.Cg-*Gt(ROSA)26Sor*^tm14(CAG-tdTomato)^Hze/J (Ai14) mice (stock no. 007914, Jackson Laboratories). All mice were group-housed (two to five per cage) in a temperature- and hygrometry-controlled room on a 12-hour/12-hour reverse light/dark cycle. For all surgeries, animals were anesthetized with an isoflurane/oxygen vapor mixture (4% for induction; 1.5 to 2% for maintenance of anesthesia) (Patterson Veterinary Supply Inc.). Intracranial virus injections were performed using a stereotaxic frame (Kopf). The following coordinates were used (from bregma and dura): mPFC: anterior-posterior (A-P), +2.0 mm; medial-lateral (M-L), ±0.8 mm; dorsal-ventral (D-V), −2.0 mm; angle 11° from midline; LHb: A-P, −1.65 mm; M-L, ±0.45 mm; D-V, −2.82 mm; VTA: A-P, −3.1 mm; M-L, ±0.6 mm; D-V, −4.75 mm; MRn: A-P, −4.2 mm; M-L, ±0.0 mm; D-V, −3.85 mm; LC: A-P, −5.1 mm; M-L, ±0.09 mm; D-V, −2.9 mm. For photometry optical fiber implants (Doric) into the mPFC, the same coordinates as above were used except that the D-V coordinate was 0.2 mm higher. Virus infusion and optical fiber implant were performed during the same intracranial surgery. Optical fibers were custom-made (Doric). After the virus infusions, the fiber was implanted and secured with two screws in dental cement. All behavioral tests were performed during the dark phase of the light/dark cycle. Animals had ad libitum access to food and water throughout the experiments except for the intravenous cocaine self-administration and operant food responding experiments, when animals were food restricted to 85 to 90% of their free-feeding body weight. During the self-administration and food responding experiments, body weights of mice were monitored daily.

### Drugs

Yohimbine (2 mg kg^−1^; Sigma-Aldrich) was dissolved in purified water. CNO (3 mg kg^−1^; Enzo Life Sciences) was dissolved in a dimethyl sulfoxide:physiological saline (5:95 ratio) solution. Cocaine was dissolved in physiological saline.

### Viruses

Depending on the experiment, the following viruses were used: AAV5-DIO-hM4Di-mCherry, AAV5-DIO-mCherry (control virus), AAV5-CAG-FLEX-Gcamp6m.WPRE.SV40 (Addgene), rgAAV-hSYN-GFP-2A-iCre, rgAAV-hSYN-fDIO-GFP-2A-iCre, and rgAAV-hSYN-eBFP-2A-FLPo (Vector Biolabs); or AAV8-CAG-FLEX-TCB (TVA), AAV8-CAG-FLEX-WPRE-SV40-oG, and RvEnvA deleted prot-G-GFP (Salk Institute). After intracranial surgeries, mice were visually inspected, and their body weight was recorded each day for seven consecutive days, after which their weight was recorded weekly. Experiments started ~28 days after surgeries to permit the mice to fully recover and to facilitate virus-mediated expression of transgenes. At the end of each experiment, brains were perfused (10 ml of 1× phosphate-buffered saline and then 15 ml of 4% paraformaldehyde), cryopreserved (48 hours, 30% sucrose), snap-frozen, and then sliced (cryostat, Leica CM 3050 S). Fluorescence was assessed under a fluorescence microscope (Zeiss). In all cases, the accuracy of injection sites was carefully confirmed, and only animals with injections located in targeted brain sites were included in statistical analyses.

### Fiber photometry

Calcium signals were collected using a Doric system at a sampling frequency of 12 kHz. We equalized the 405- and 465-nm signals to record an equivalent signal/noise ratio. Custom-generated MATLAB scripts were used to down-sample and normalize the fluorescence signal. The fluorescence from the control channel (*F*_405_; isobestic point) was filtered using a polyfit regression giving a fitted control (*F*_405c_). Δ*F*/*F* was calculated as (*F*_465_ − *F*_405c_)/*F*_405_. The deviation of each sample from the averaged signal of a given period was calculated with a *z* score. Recording sessions were conducted in the same chambers used for fear conditioning experiments, with 3 min of preshock baseline recordings conducted immediately before delivery of noxious electrical foot shock (0.6 mA, 2 s), followed by 3 min of postshock recordings.

### Patch-clamp recordings

Mice were deeply anesthetized with isoflurane and then decapitated. Brains were rapidly removed and chilled in “cutting” artificial cerebrospinal fluid (aCSF) containing the following: 93 mM *N*-methyl-d-glucamine, 93 mM HCl, 2.5 mM KCl, 1.2 mM NaH_2_PO_4_, 30 mM NaHCO_3_, 20 mM Hepes, 25 mM glucose, 5 mM sodium ascorbate, 2 mM thiourea, 3 mM sodium pyruvate, 10 mM MgSO_4_, and 0.5 mM CaCl_2_ (pH 7.4). The brain was embedded in 2% agarose, and coronal slices (300 μm thick) were made using a Compresstome (Precisionary Instruments). Brain slices were allowed to recover for 30 min at 33° ± 1°C in the cutting solution and then incubated for ~60 min at room temperature in “holding” aCSF solution containing the following: 92 mM NaCl, 2.5 mM KCl, 1.2 mM NaH_2_PO_4_, 30 mM NaHCO_3_, 20 mM Hepes, 25 mM glucose, 5 mM sodium ascorbate, 2 mM thiourea, 3 mM sodium pyruvate, MgSO_4_, and 2 mM CaCl_2_ (pH 7.4). Slices were then transferred to a submersion recording chamber and continuously perfused (2 to 4 ml min^−1^) with aCSF containing the following: 124 mM NaCl, 2.5 mM KCl, 1.2 mM NaH_2_PO_4_, 24 mM NaHCO_3_, 5 mM Hepes, 12.5 mM glucose, 2 mM MgSO_4_, and 2 mM CaCl_2_ (pH 7.4). All solutions were continuously bubbled with 95% O_2_/5% CO_2_. mCherry-expressing cells in the mPFC were visually identified with fluorescent/infrared differential contrast optics (BX51, Olympus). Whole-cell patch-clamp recordings were performed at room temperature using a MultiClamp 700A amplifier (Molecular Devices). Recording electrodes (3 to 5 megohms) pulled from borosilicate glass were filled with solution containing the following: 115 mM K-gluconate, 10 mM Hepes, 20 mM KCl, 1.5 mM MgCl_2_, 2 mM MgATP, 0.5 mM Na_2_GTP, 10 mM Na-phosphocreatine, and 0.1 mM EGTA (pH 7.25). Data acquisition (filtered at 10 kHz and digitized at 10 kHz) and analysis were performed with pClamp11 software (Molecular Devices).

### MAPseq connectome profiling

We performed MAPseq according to the published protocol from Kebschull and co-workers (*54*). Neurons were randomly labeled with genetically encoded barcodes from a library of virus particles. Ideally, every infected neuron would have a single, unique barcode (*54*). This was accomplished by injecting the MAPseq virus (Sindbis virus, Cold Spring Harbor Laboratories) into the mPFC. Forty-eight hours later, brains were removed, snap-frozen, and then cut into 300-μm coronal sections using a cryostat (Leica CM 3050 S). Brain areas of interest were dissected on dry ice, and total RNA was extracted from each sample. A gene-specific reverse transcription reaction for the barcode mRNA was performed; double-stranded complementary DNA was generated and amplified by polymerase chain reaction to produce an Illumina sequencing library. This library was then sequenced by the Cold Spring Harbor Laboratories MAPseq core accordingly to previously reported methods (*54*) on an Illumina NextGen sequencing machine. Raw MAPseq data consisted of two Fastq files containing Illumina sequencing results, where paired end 1 covers the barcode sequence, and paired end 2 covers the 12-nt unique molecular identifier and the 6-nt slice-specific identifier. To convert these sequencing data into projection maps, the data were first preprocessed in bash, before being analyzed in MATLAB (MathWorks). We processed the sequencing data to determine the exact copy number of each barcode sequence in each target area and in the injection site. We then produced a barcode matrix where each row corresponds to one specific barcode sequence, each column corresponds to a target area or the injection site, and each entry is the copy number of that barcode mRNA in the respective area. Using a custom-made MATLAB script, we isolated each barcode in each brain area of interest and calculated the percentage of reads for each barcode relative to the total; the number of rads for the same barcode in the virus injection site to generate a measure of relative projection strength: [(number of barcode observed in a brain area of interest/all the barcode detected in the injection site)*100]. Then, for each brain area, we also calculate the percentage of barcode observed in every other brain area dissected.

### Operant responding for food rewards

Mice were mildly food restricted to 85 to 90% of their free-feeding body weight and trained to press an active lever in an operant chamber (Med Associates) to earn food rewards (20 mg of chow pellets; TestDiet) under an FR5TO20 schedule of reinforcement during 60-min daily sessions (~5 days/week). During each session, an inactive lever was also extended into the chamber, responding on which was recorded but was without scheduled consequence. Mice were permitted to respond until they demonstrated stable responding for food rewards (>20 pellets earned during each session). For chemogenetic manipulations, CNO was injected 5 min before placing the animals in the operant chambers for the 60-min test session. For the behavioral flexibility assessment, the active and inactive lever assignments were switched immediately before the session.

### Intravenous cocaine self-administration, extinction, and reinstatement

Mice were mildly food restricted and trained to press an active lever for food pellets under an FR5TO20 schedule of reinforcement as described above. Once they demonstrated stable responding for food rewards, they were surgically prepared with chronically indwelling intravenous jugular catheters. Briefly, each mouse was anesthetized with an isoflurane (1 to 3%)/oxygen vapor mixture, and a catheter was inserted 1 cm into the right jugular vein and secured with surgical silk suture. The catheter consisted of Silastic tubing, 6 cm in total length, that was fitted to guide a cannula (Plastics One) bent at a curved right angle and encased in dental acrylic. The catheter tubing was passed subcutaneously from the jugular vein to an exit port on the animal’s back. Catheters were flushed daily with physiological sterile saline solution [0.9% (w/v)] containing heparin (10 to 60 USP units ml^−1^). Catheter integrity was tested with the ultrashort-acting barbiturate anesthetic Brevital (methohexital sodium; Eli Lilly).

Mice were permitted at least 48 hours to recover from catheter surgery and then permitted to respond for food rewards again under the same FR5TO20 schedule. Once stable food responding was reestablished, subjects were permitted to respond for intravenous cocaine infusions (0.3 mg kg^−1^ per infusion) via the chronically implanted jugular catheter. Self-administration sessions were 60 min and were conducted 7 days/week. Each self-administration session commenced by the extension of a drug-paired active lever and an inactive lever into the chamber. Completion of the response criteria on the active lever resulted in the illumination of a cue light located immediately above the active lever, which coincided with the delivery of the intravenous cocaine infusion and the initiation of the 20-s time-out period. To examine the effects of chemogenetically inhibiting cortico-habenular neurons on cocaine self-administration behavior, we injected mice with vehicle or CNO (3 mg kg^−1^) 5 min before placing them in chamber for a 60-min self-administration session. For all sessions, numbers of active and inactive lever presses and cocaine infusions earned were recorded.

To extinguish cocaine-seeking responses, we rendered both levers inactive such that responding on either had no scheduled consequence during the 60-min session. Mice demonstrated reliably reduced responding on the previously active lever after ~14 daily extinction sessions. To examine the effects of chemogenetically inhibiting cortico-habenular neurons on extinction of cocaine self-administration behavior, we injected mice with vehicle or CNO (3 mg kg^−1^) 5 min before placing them in chamber for a 60-min extinction session. Numbers of responses on the previously active and inactive levers were recorded. Once responding had fully extinguished, we examined the effects of yohimbine injection or exposure to the cocaine-paired cue light on responding on the previously active and inactive levers. In the case of yohimbine-precipitated reinstatement, mice were injected with vehicle or CNO 10 min before the start of the reinstatement session and then injected with yohimbine (2 mg kg^−1^) 5 min later. Responding on the previously active and inactive levers was recorded for 60 min, with responding on either lever having no scheduled consequence. Responding on the previously active and inactive levers was then permitted to extinguish again during daily extinction sessions. In the case of cue-precipitated reinstatement, mice were injected with vehicle or CNO 10 min before the start of the reinstatement session and then placed into the operant chamber. The previously active and inactive levers were extended into the chamber, the cocaine-paired cue light was transiently activated (20 s), and then lever-pressing behavior was recorded for 60 min. Completion of each FR5 schedule on the active lever activated the cocaine-paired cue light for 20 s.

### Conditioned place preference

CPP was performed using 10 identical CPP chambers composed of two compartments (each with 20 cm by 20 cm dimensions). To facilitate discrimination between the compartments, each contained different visual cues and different floor textures. Mice were habituated to the apparatus before commencing the conditioning sessions. Specifically, they were permitted to freely explore the apparatus for 15 min on day 1, and this habituation procedure was repeated on the subsequent day. We then calculated a baseline preference score based on day 2 performances [(time spent in each compartment/total time)*100]. On conditioning days (days 3 to 7), mice received a saline injection in the morning (a.m. session) and were restricted to the saline-paired compartment for 15 min. The compartment that mice had preferred during the habituation session (>50% time spent in that compartment) was designated as the saline-paired compartment in all cases (i.e., “biased” CPP protocol). On the same day (∼4 hours later; p.m. session), mice were injected with cocaine (20 mg kg^−1^) and confined to the other “cocaine-paired” compartment for 15 min. On the test day (day 8), conducted after 5 days of conditioning, mice were injected with saline and, 15 min later, placed into the CPP apparatus and permitted to freely explore freely. The time, distance, and resting time spent in each compartment were automatically recorded using environmental control chambers (Fusion software, Omnitech Electronics Inc.). Preference scores were calculated according to the following formula: [(total time in the cocaine-paired side/total time spent in both compartments)*100].

For yohimbine-induced reinstatement, mice underwent an extinction period consisting of 15-min daily sessions under treatment-free conditions when they were permitted to freely explore the apparatus (as during the habituation phase and CPP test). When preference for the cocaine-paired chamber was extinguished, which usually required three to four extinction sessions depending on the cohort, the yohimbine-induced reinstatement phase commenced. Mice were injected with yohimbine (2 mg kg^−1^) or saline (control) 5 min before being placed in the apparatus for 15 min, during which they could explore freely. Time, distance, and resting time spent in each compartment were automatically recorded, and chamber preference scores were calculated as described above. CNO injections occurred 15 min before test sessions (i.e., 10 min before yohimbine or saline injection).

### Locomotor activity and anxiety assessments

Locomotor activity was assessed in an open-field apparatus (40 cm by 40 cm; Omnitech Electronics Inc.). For chemogenetic manipulations, CNO was injected 15 min before the start of the test session, then mice were placed in the center of the open field apparatus, and their behavior was monitored for 30 min. Total distance traveled, time spent inactive (resting time), time spent along the edges of the apparatus (thigmotaxis), and time spent in the center of the apparatus were automatically recorded (Fusion software, Omnitech Electronics Inc.). Anxiety-related behaviors were assessed using a light-dark box test. The apparatus contained two interconnected compartments, each of which had dimensions of 20 cm by 20 cm (Omnitech Electronics Inc.). One of the compartments was illuminated during the test session (“light” side), and the other was unilluminated (“dark” side). Fifteen minutes before the test session, mice were injected with CNO, and then during the test session, they were permitted to freely explore the apparatus for 30 min. The time, distance, and resting time spent in each compartment were automatically recorded. Percentages of time spent in each side were then calculated as [(time in light side/total time)*100].

### Fear conditioning

Fear conditioning was assessed in four conditioning chambers (17 cm by 17 cm by 25 cm) with electrified grid floors; each one was located in a sound-attenuating box, with ventilating fan, a light, a speaker, and a USB camera (Ugo Basile). The protocol lasted for 2 days: For day 1 (conditioning), 15 min after CNO or saline injection, mice were exposed to the conditioning chambers. After a 3-min baseline period with no scheduled events, mice were exposed to three consecutive conditioned stimulus (CS)-unconditioned stimulus (US) presentations (CS: 30-s tone, 60 dB, 3000 Hz; US: 0.6 mA of electrical foot shock for 2 s, with a 1 min ± 30 s interval; CS lasted for 30 s, and the US was triggered 2 s before the end of the CS). For day 2 (context and cue test): context test: 15 min after CNO or vehicle injection, mice were placed in the same chamber as in day 1 for 5 min; cue test: 60 min after the context test, mice were injected with CNO or vehicle again and, 15 min later, exposed to a new and different context for 6 min. After a 3-min baseline period with no scheduled events, the CS was turned on for 3 min. Times spent immobile and distances traveled were automatically recorded (Noldus, EthoVision). The percentage of freezing [(time of immobility/total time)*100] was then calculated.

### Statistics

All data were analyzed by one-way analysis of variance (ANOVA) or *t* test using GraphPad Prism version 9.0.0 for Windows (GraphPad Software, San Diego, CA, USA). One-sample *t* tests were used to analyze CPP data, with proportions of times spent on either side of the apparatus compared to a hypothetical proportion of time (50%). During reinstatement tests, we also compared the mean time spent on the cocaine-paired side between groups by unpaired two-tailed *t* tests. Significant main or interaction effects in ANOVAs were followed by Bonferroni or Newman-Keuls post hoc tests as appropriate. The criterion for significance was set at *P* < 0.05. All results are represented as the means ± SEM.

## Notes

View/request a protocol for this paper from *Bio-protocol*.
