# Supplementary material for: Networks of habenula-projecting cortical neurons regulate cocaine seeking
Source: Sci Adv. 2021 Nov 5;7(45):eabj2225. doi: 10.1126/sciadv.abj2225 (PMC8570600; doi:10.1126/sciadv.abj2225)
Supplement: Supplementary file 1 — Figs. S1 to S17 [file sciadv.abj2225_sm.pdf]

Science Advances
AAAS

Supplementary Materials for

Networks of habenula-projecting cortical neurons regulate cocaine seeking

Victor P. Mathis*, Maya Williams, Clementine Fillinger, Paul J. Kenny*

*Corresponding author. Email: paul.kenny@mssm.edu (P.L.); victor.mathis@dbmail.com (V.M.)

Published 5 November 2021, *Sci. Adv.* **7**, eabj2225 (2021)
DOI: 10.1126/sciadv.abj2225

This PDF file includes:

Figs. S1 to S17

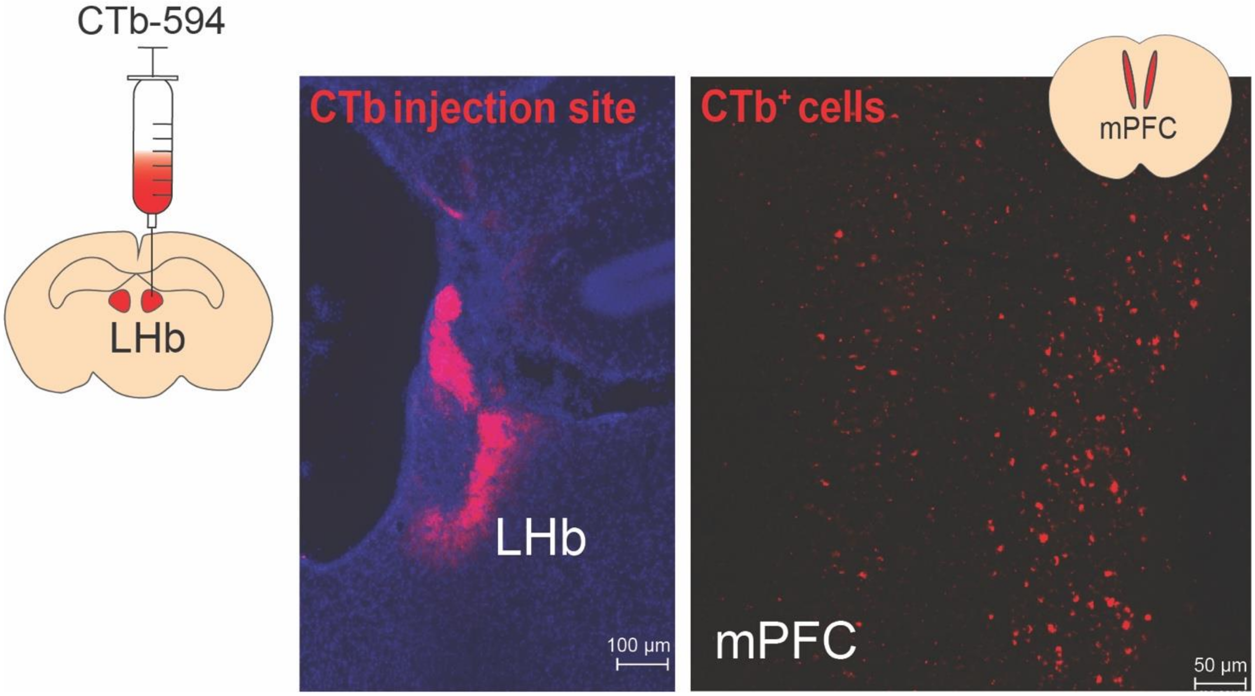

Fig. S1. Cortical projection to the lateral habenula. Graphical representation of intracranial injection procedure to label cortico-habenular neurons (Left panel). Representative fluorescence from tdTom$^+$ neurons in LHb after local injection CTB-594 (middle panel). Representative tdTom$^+$ cells in mPFC of mice that received intra-LHb injection of CTB-594 (right panel). A total of n=5 mice were imaged.

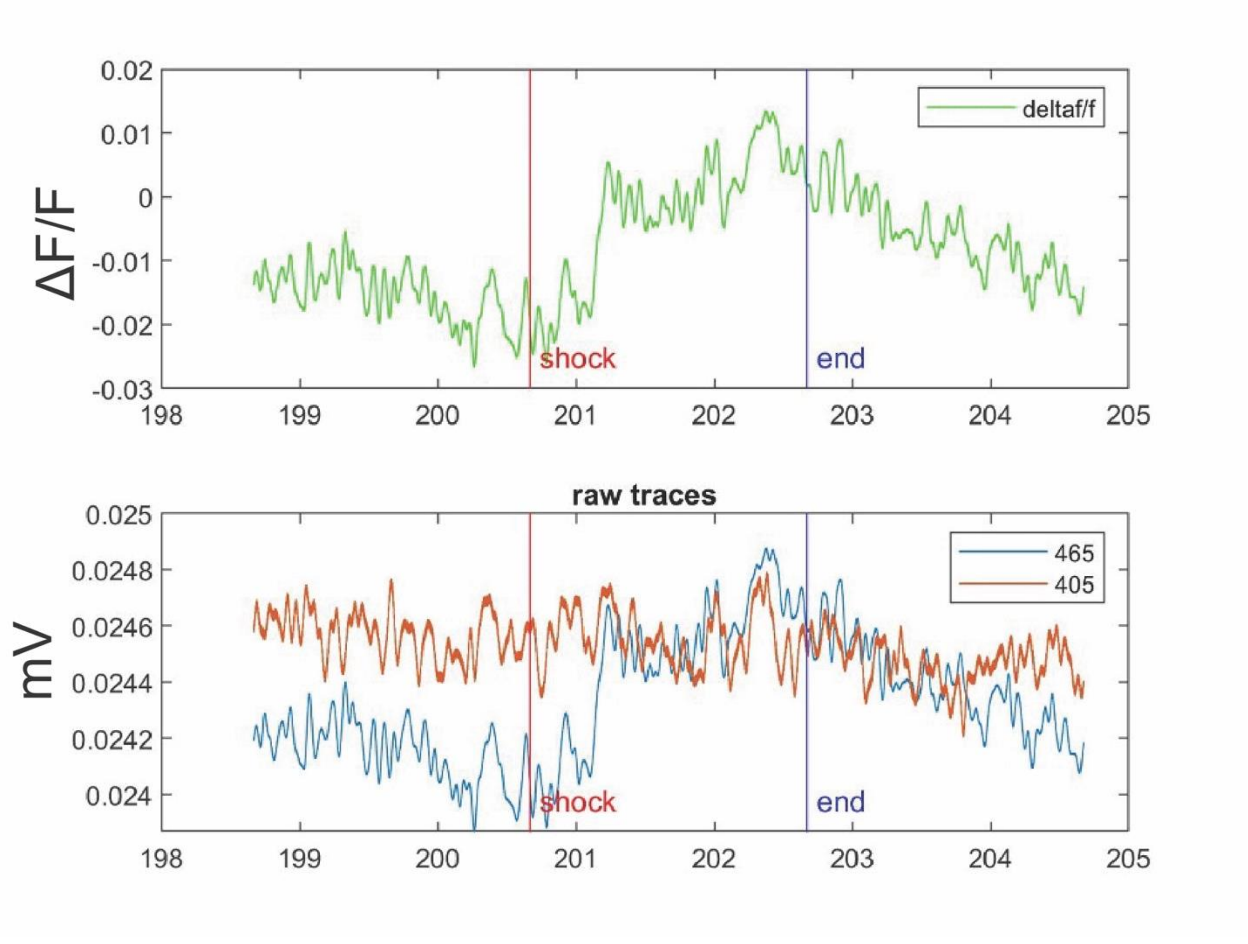

Fig. S2. Fiber photometry of mPFC$_{\rightarrow LHb}$ neurons. Representative raw traces from the 465 nm and 405 nm (isobestic point) channels. A total of *n*=3 mice were recorded.

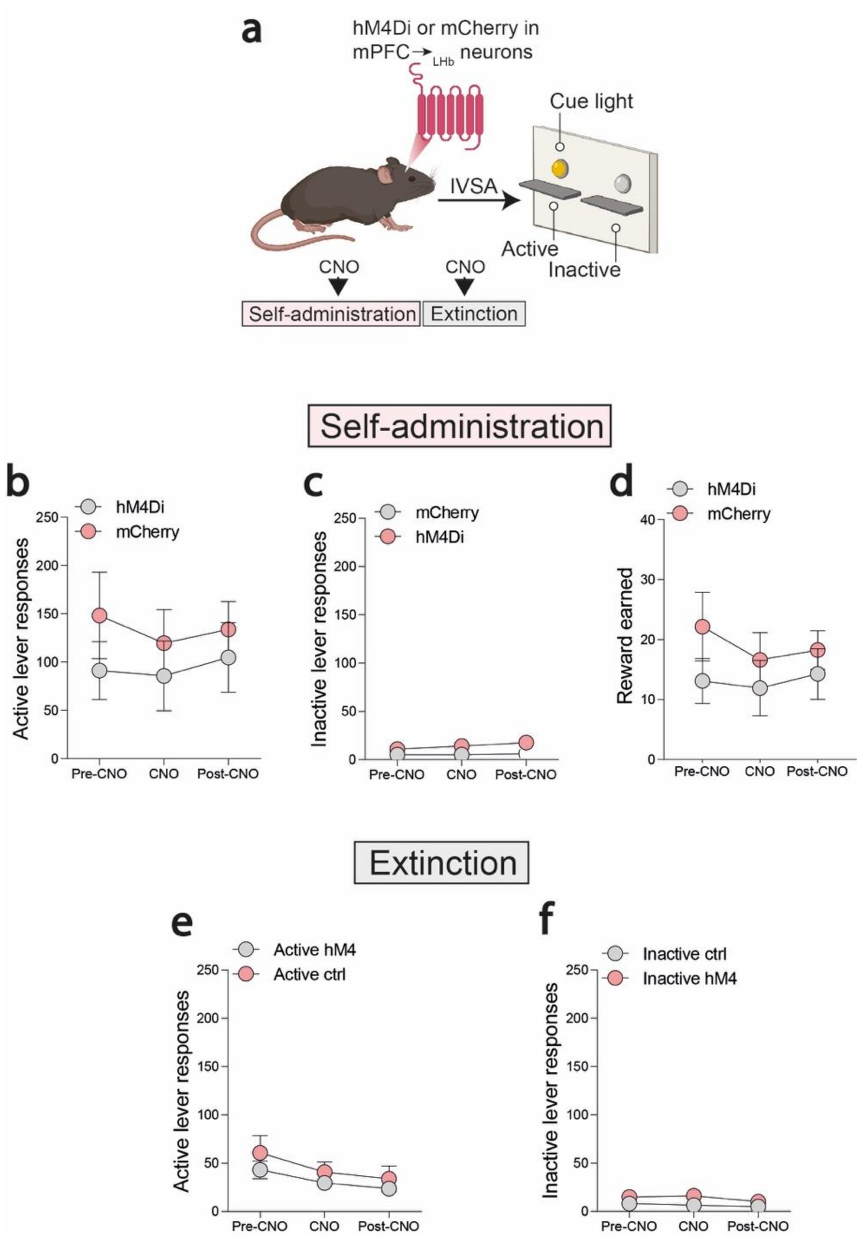

Fig. S3. mPFC$_{\rightarrow LHb}$ neurons do not regulate cocaine reinforcement or extinction. (**a**) Graphical representation of the IV self-administration (IVSA) procedure. (**b**) Active lever press responses by mice expressing hM4Di (*n*=11) or mCherry (*n*=7) in cortico-habenular neurons responding for cocaine rewards (0.3 mg kg^{-1} per infusion) during the session before CNO injection (Pre-CNO), during the CNO treatment session (3 mg kg^{-1}; injected 5 min before initiation of session), and during the session conducted on the day after CNO treatment (Post-CNO); Two-way ANOVA: *DREADD* $F_{(1,16)}$=0.625, *p*=0.441; *Session* $F_{(2,32)}$=1.739, *p*=0.205; *DREADD* x *Session* $F_{(2,32)}$=1.022, *p*=0.371. (**c**) Inactive lever presses in hM4Di and mCherry mice during the Pre-CNO, CNO and Post-CNO sessions. Two-way ANOVA: *DREADD* $F_{(1,16)}$=2.179, *p*=0.159; *Session* $F_{(2,32)}$=1.368, *p*=0.266; *DREADD* x *Session* $F_{(2,32)}$=0.690, *p*=0.509. (**d**) Number of cocaine rewards earned by hM4Di and mCherry mice during the Pre-CNO, CNO and Post-CNO sessions. Two-way ANOVA: *DREADD* $F_{(1,16)}$=1.216, *p*=0.287; *Session* $F_{(2,32)}$=1.572, *p*=0.228; *DREADD* x *Session* $F_{(2,32)}$=0.774, *p*=0.470. (**e**) Active lever presses under extinction conditions in hM4Di and mCherry mice during the Pre-CNO, CNO and Post-CNO extinction sessions. Two-way ANOVA: *DREADD* $F_{(1,16)}$=1.155, *p*=0.159; *Session* $F_{(3,32)}$=5.350, *p*=0.0137; *DREADD* x *Session* $F_{(2,32)}$=0.1496, *p*=0.8617. (**f**) Inactive lever presses under extinction conditions in hM4Di and mCherry mice during the Pre-CNO, CNO and Post-CNO extinction sessions. Two-way ANOVA: *DREADD* $F_{(1,16)}$=1.707, *p*=0.159; *Session* $F_{(2,32)}$=1.813, *p*=0.1831; *DREADD* x *Session* $F_{(2,32)}$=0.5015, *p*=0.6103.

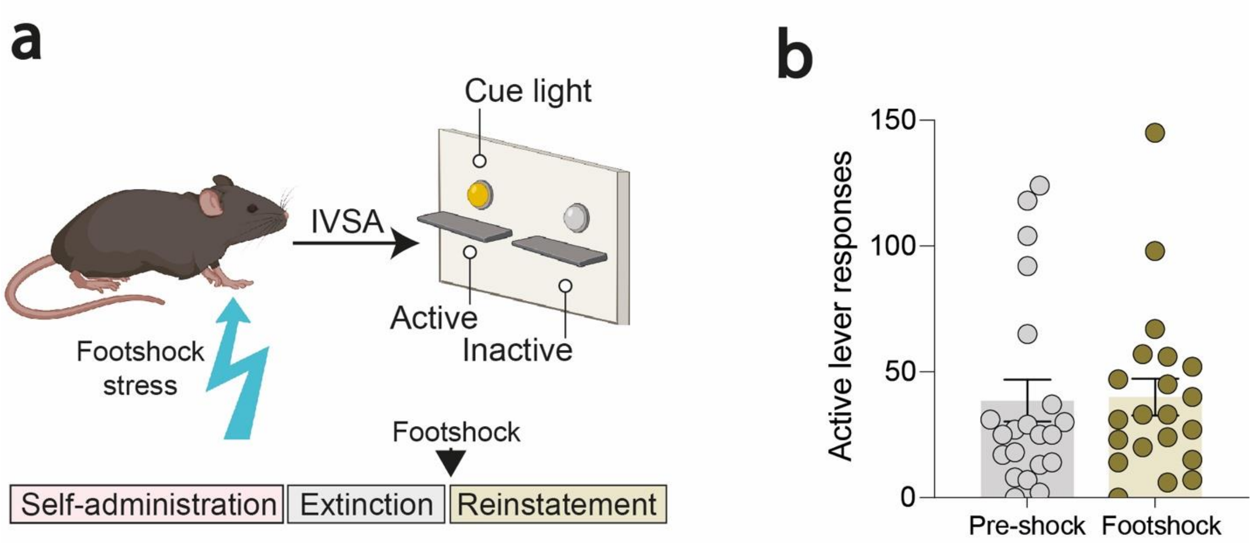

Fig. S4. Electrical footshock does not induced reinstatement in mice. (**a**) Graphical representation of the footshock-induced reinstatement procedure. The footshock parameters were: 5 min exposure prior to reinstatement session; 1 mA intensity; 0.5 sec on; mean interval between shocks was 40 sec; range of intervals between shocks was 10–70 sec. (**b**) Active lever press responses under extinction conditions during the session preceding (Pre-shock) and the session immediately after footshock stress (0.4 mA) in mice that had stably responded for cocaine infusions under a FR5TO20 sec schedule of reinforcement but in which responding had been extinguished (*n*=21); t-test$_{(40)}$=0.125, *p*=0.901. All data are expressed as mean (± SEM) number of lever presses.

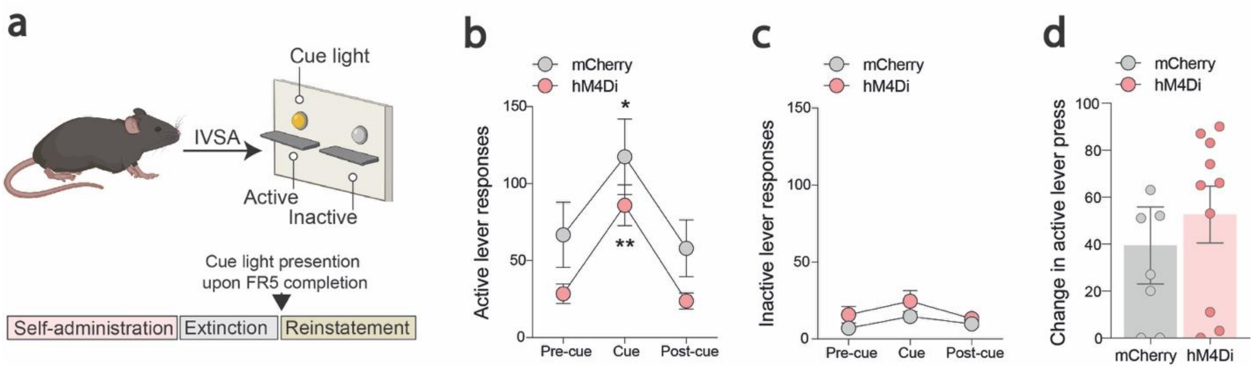

Fig. S5. mPFC$_{\rightarrow \text{LHb}}$ neurons do not regulate cue-induced reinstatement. (**a**) Graphical representation of the cue-induced reinstatement procedure. (**b**) Active lever press responses during the daily extinction session immediately preceding (Pre-cue), during the reinstatement session (Cue), and during the session after (Post-cue) cue-induced reinstatement of cocaine seeking in mice expressing hM4Di (*n*=10) or mCherry (*n*=7) in cortico-habenular neurons.Two-way ANOVA: *DREADD* $F_{(1,16)}$=3.883, *p*=0.066; *Session* $F_{(2,32)}$=27.69, *p*<0.0001; *DREADD* x *Session* $F_{(2,32)}$=0.070, *p*=0.932. ***P*<0.01, **p*<0.05 on Cue session compared with Pre-cue session in same group of mice; Tukey post-hoc test. (**c**) Inactive lever press during the Pre-cue, Cue, and Post-cue sessions. Two-way ANOVA: *DREADD* $F_{(1,16)}$=1.258, *p*=0.2786; *Session* $F_{(2,32)}$=3.629; *DREADD* x *Session* $F_{(2,32)}$=0.488, *p*=0.618. (**d**) Change in number of active lever presses during the Cue session compared with Pre-cue session; t-test$_{(19);}$ *p*=0.661. All data are expressed as mean (± SEM) number of lever presses.

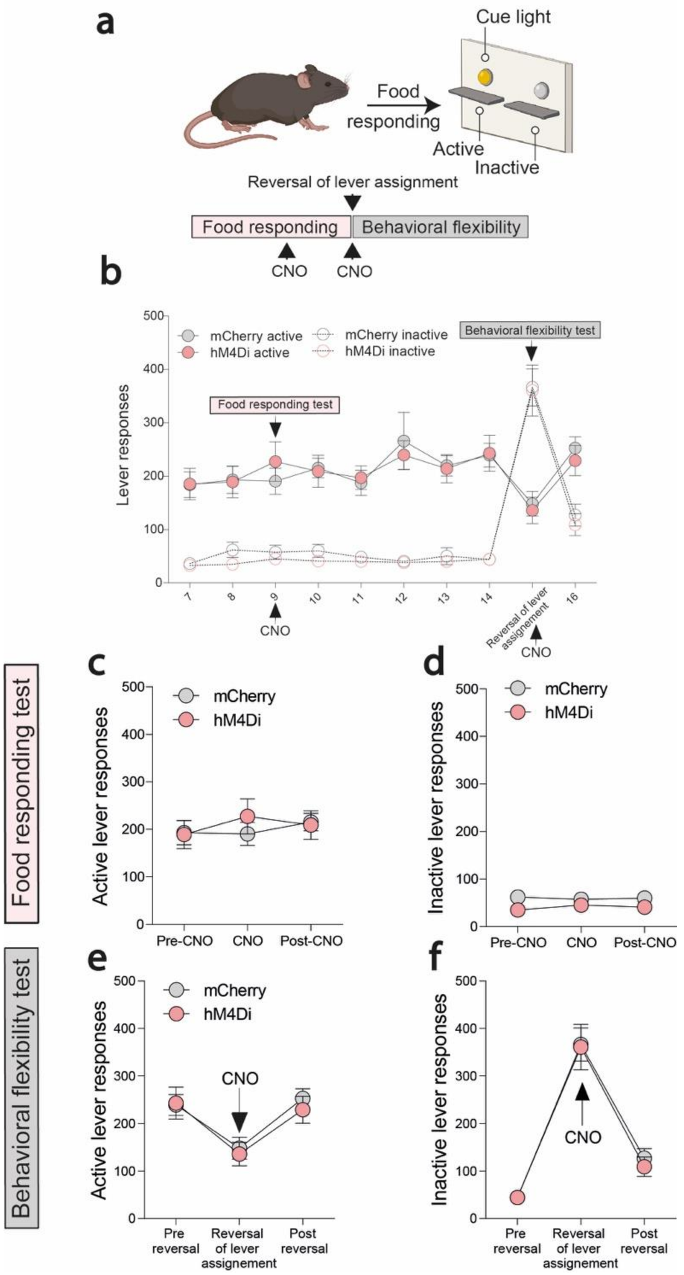

Fig. S6. mPFC→LHb neurons do not regulate food responding or behavioral flexibility. (**a**) Graphical representation of procedure used to investigate the role for cortico-habenular neurons in operant responding for food reinforcers and behaviour flexibility. (**b**) Active and inactive levers responses to earn food rewards (25 mg pellets) under a FR5TO20 sec reinforcement schedule across daily sessions. Mice (hM4Di *n*=15; mCherry *n*=10) were injected with CNO prior to sessions identified by black arrows. (**c**) Active lever responses during the daily session preceding (Pre-CNO), on the test day (CNO), and during the session after (Post-CNO) testing the effects of CNO on food responding. Two-way ANOVA: *DREADD* $F_{(1,23)}=0.044$, $p=0.835$; *Session* $F_{(2,46)}=1.937$, $p=0.157$; *DREADD* x *Session* $F_{(2,46)}=2.204$, $p=0.122$. (**d**) Inactive lever responses during the Pre-CNO, CNO and Post-CNO sessions. Two-way ANOVA: *DREADD* $F_{(1,23)}=2.065$, $p=0.164$; *Session* $F_{(2,46)}=0.121$, p=0.849; *DREADD* x *Session* $F_{(2,46)}=0.727$, $p=0.489$. (**e**) Active lever responses during the daily session preceding (Pre-CNO), on the test day (CNO), and during the session after (Post-CNO) testing the effects of reversing the lever assignments on food responding. Main effect of *Session* in two-way ANOVA: $F_{(2,46)}=20.58$, $p<0.0001$. (**f**) Inactive lever responses during the Pre-CNO, CNO and Post-CNO sessions. Main effect of *Session* in two-way ANOVA: *Session* $F_{(2,46)}=71.17$, $p<0.0001$. All data are expressed as mean (± SEM) number of lever presses.

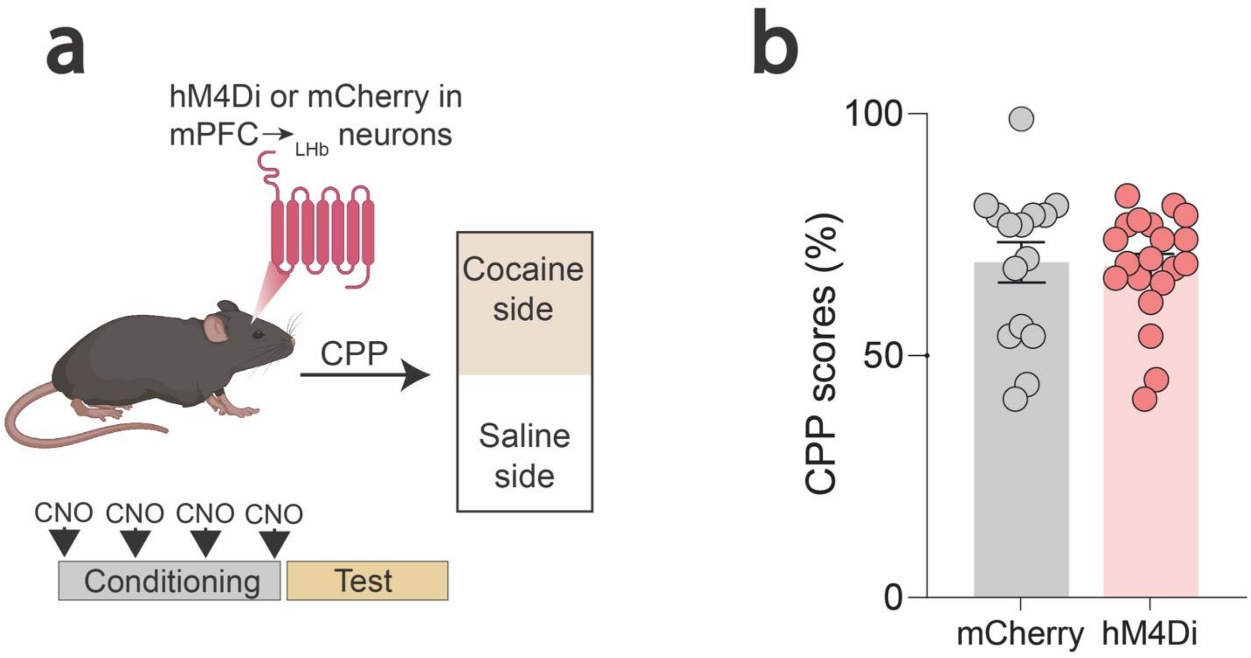

Fig. S7. mPFC→LHb neurons do not regulate cocaine reward. (**a**) Graphical representation of the CPP apparatus and experimental design to investigate the involvement of cortico-habenular neurons in cocaine reward. (**b**) CPP scores (percentage of total time spent in cocaine-paired side) on the test day for mice expressing hM4Di (*n*=20) or mCherry (*n*=15) in cortico-habenular neurons after 5 daily cocaine conditioning sessions. Mice were injected with CNO prior to each conditioning session but were not injected before the test session. One-sample t-test: $t_{(14)}=4.625$, $p=0.0004$ for mCherry mice; $t_{(19)}=7.374$, $p<0.0001$ for hM4Di mice. Unpaired two-sided t-test: t-test: $t_{(33)}=0.155$, $p=0.878$ for mCherry *vs.* hM4Di mice. All data are expressed as mean (± SEM) percentage of time spent on the cocaine-paired side of the apparatus.

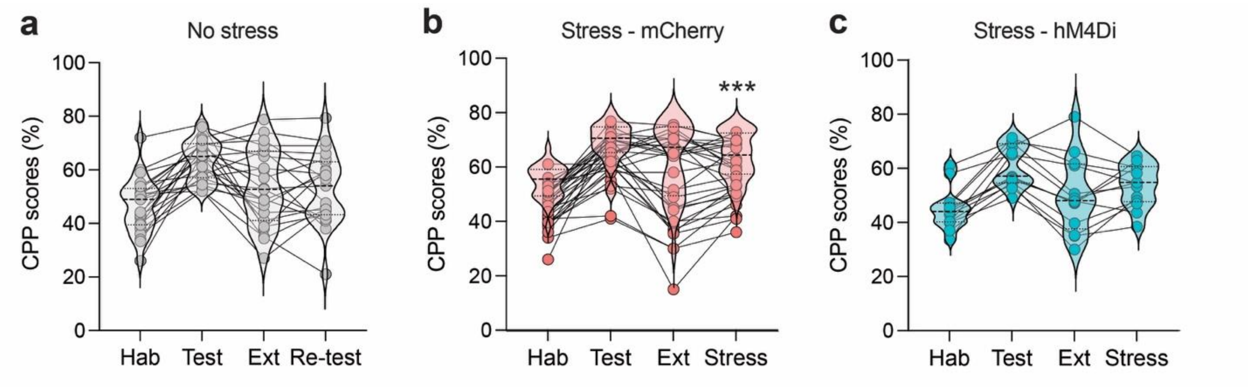

Fig. S8. mPFC$_{\rightarrow LHb}$ neurons regulate cocaine seeking. (**a**) Mean (± SEM) percentage of time spent in the cocaine-paired side of the CPP apparatus during the habituation (Hab), preference test for cocaine-paired side (Test), and after extinction sessions (Ext) in mice expressing hM4Di in cortico-habenular neurons and injected with CNO then vehicle prior to the re-test session. (**b**) Mean (± SEM) percentage of time spent in the cocaine-paired side of the CPP apparatus during the habituation, test session, and after extinction sessions in mice expressing mCherry in cortico-habenular neurons and injected with CNO prior to yohimbine injection during the 'stress' session. ***P<0.001, one-sample t-test, actual mean compared with theoretical mean during the 'Stress' session. (**c**) Mean (± SEM) percentage of time spent in the cocaine-paired side of the CPP apparatus during the habituation, preference test, and after extinction sessions in mice expressing hM4Di in cortico-habenular neurons and injected with CNO prior to yohimbine injection during the 'stress' session.

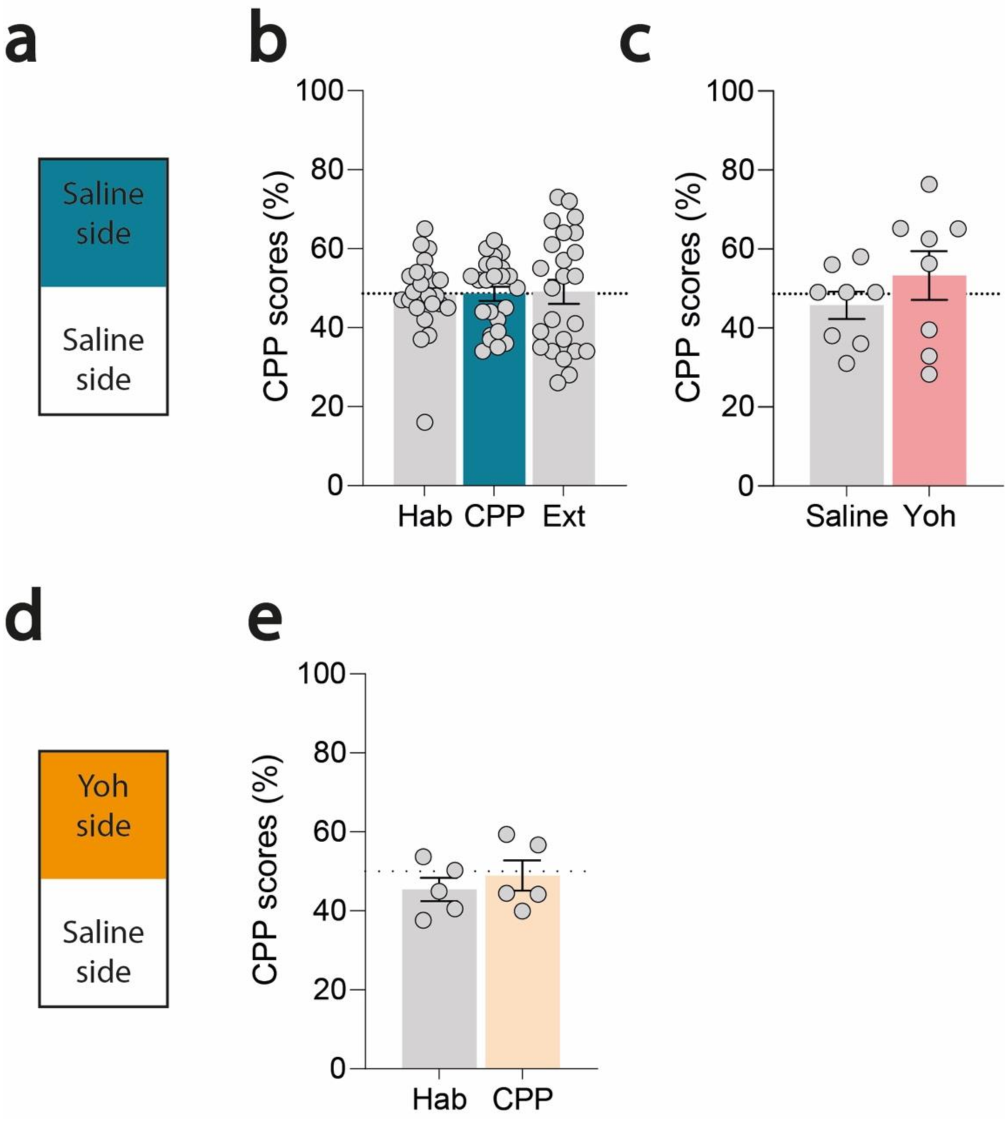

Fig. S9. Saline or yohimbine injections do not condition a place preference. (a) Graphical representation of the CPP protocol. During conditioning session, saline injections were paired with both sides of the apparatus. (**b**) CPP scores during the habituation (Hab), saline conditioning (CPP) and after the extinction phase (Ext) phase of the experiment. After conditioning, mice (*n*=24) did not express preference for either side of the apparatus. One sample t-test for Hab $t_{(23)}$=0.848, *p*=0.405; CPP $t_{(23)}$=0.795, *p*=0.435; Ext $t_{(23)}$=0.302, *p*=0.766. (**c**) Yohimbine injection did not induce any change in preference for either side of the CPP apparatus in saline-conditioned mice. One sample t-test for Saline injection $t_{(7)}$=1.238, *p*=0.256; Yohimbine injection: $t_{(7)}$=0.532, *p*=0.612); unpaired two-tailed t-test for Saline *vs.* Yohimbine injection $t_{(14)}$=1.066, *p*=0.305. **(d**) Graphical representation of the CPP procedure to investigate whether yohimbine (Yoh) injections during conditioning sessions supported a change in preference. (**e**) Mice (*n*=5) did not express a preference or aversion for the yohimbine-paired side. One sample t-test: Hab $t_{(4)}$=1.537, *p*=0.199; CPP $t_{(4)}$=0.277, *p*=0.796. All data are expressed as mean (± SEM) percentage of time spent on the side of the apparatus paired with injection of the test substance.

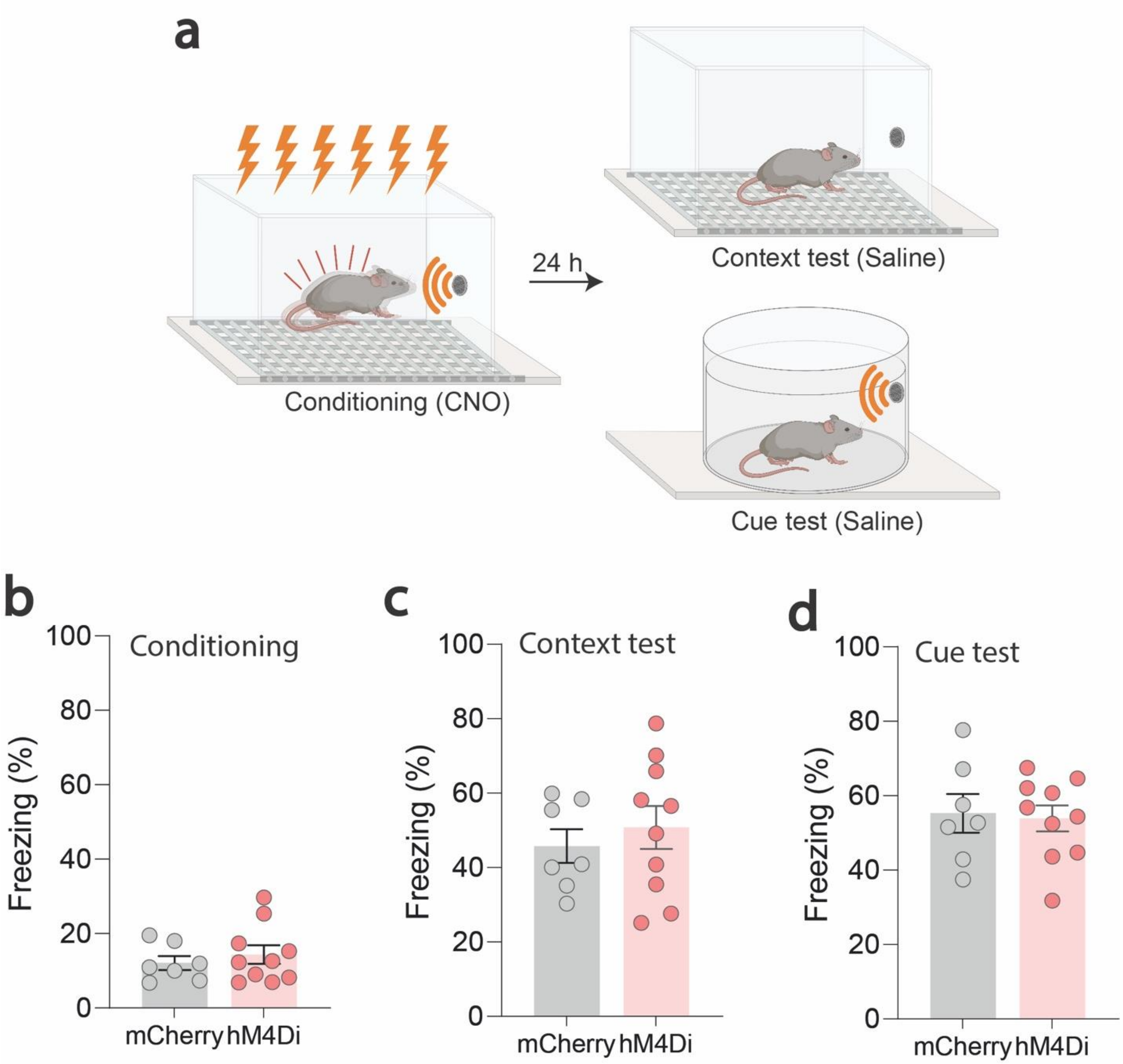

Fig. S10. mPFC$_{\rightarrow LHb}$ neurons do not regulate the encoding of conditioned fear memories. (**a**) Graphical representation of the fear conditioning procedure. Mice expressing mCherry (*n*=7) or hM4Di (*n*=10) in cortico-habenular neurons were injected with CNO prior to the fear conditioning session during which noxious footshocks were delivered in a test apparatus in conjunction with a sound cue. 24 h later, conditioned freezing was assessed in untreated mice upon exposure to the same testing apparatus (context-induced freezing) or upon exposure to the footshock-paired sound cue in a novel environment (cue-induced freezing). (**b**) Freezing responses were similar in both groups during the conditioning session. Unpaired two-tailed t-test: $t_{(15)}$=0.674, *p*=0.511. (**c**) Context-induced freezing was similar in both groups. Unpaired two-tailed t-test: $t_{(15)}$=0.637, *p*=0.534. (**d**) Cue-induced freezing was similar in both groups. Unpaired two-tailed t-test: $t_{(15)}$=0.235, *p*=0.817. All data are expressed as mean (± SEM) percentage of time spent freezing.

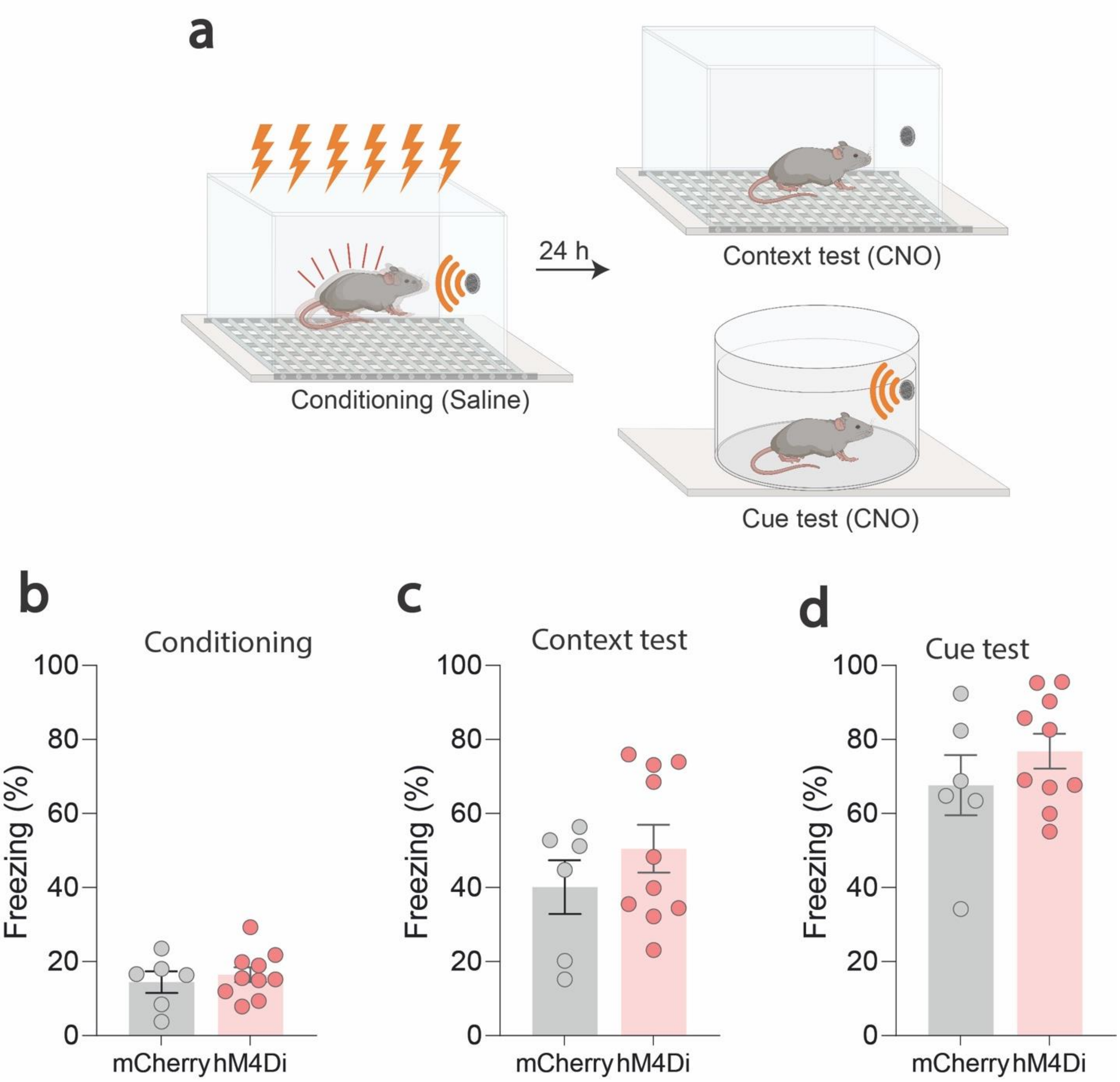

Fig. S11. mPFC$_{\rightarrow LHb}$ neurons do not regulate the expression of conditioned fear memories. (**a**) Graphical representation of the fear conditioning procedure. Mice expressing mCherry (*n*=6) or hM4Di (*n*=10) in cortico-habenular neurons were trained in the fear conditioning procedure during which noxious footshocks were delivered in a test apparatus in conjunction with a sound cue. 24 h later, mice were injected with CNO and their context- and cue-induced freezing behavior was assessed. (**b**) Freezing responses were similar in both groups during the conditioning session. Unpaired two-tailed t-test: $t_{(14)}$=0.591, *p*=0.564. (**c**) Context-induced freezing was similar in both groups. Unpaired two-tailed t-test: $t_{(14)}$=1.033, *p*=0.319. (**d**) Cue-induced freezing was similar in both groups. Unpaired two-tailed t-test: $t_{(14)}$=1.056, *p*=0.309. All data are expressed as mean (± SEM) percentage of time spent freezing.

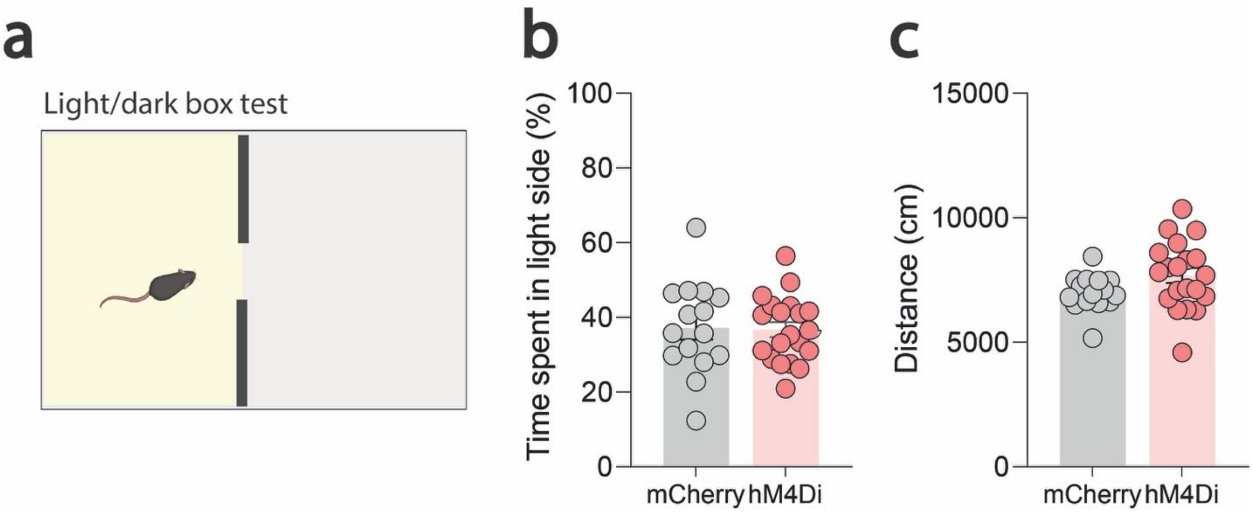

Fig. S12. mPFC$_{\rightarrow LHb}$ neurons do not regulate anxiety-related behaviors. (**a**) Graphical representation of the light-dark box procedure. Mice expressing mCherry (*n*=15) or hM4Di (*n*=20) in cortico-habenular neurons were injected with CNO then allowed to freely explore the light-dark box apparatus for 30 min. (**b**) Time spent in the light side of apparatus did not differ between groups. Unpaired two-tailed t-test: $t_{(33)}=0.138$, $p=0.891$. Data are expressed as mean (± SEM) percentage of time spent in the light side of the apparatus. (**c**) Distance travelled in the light-dark box did not differ between groups. Unpaired two-tailed t-test: $t_{(33)}=1.761$, $p=0.088$. Data are expressed as mean (± SEM) total distance (cm) travelled.

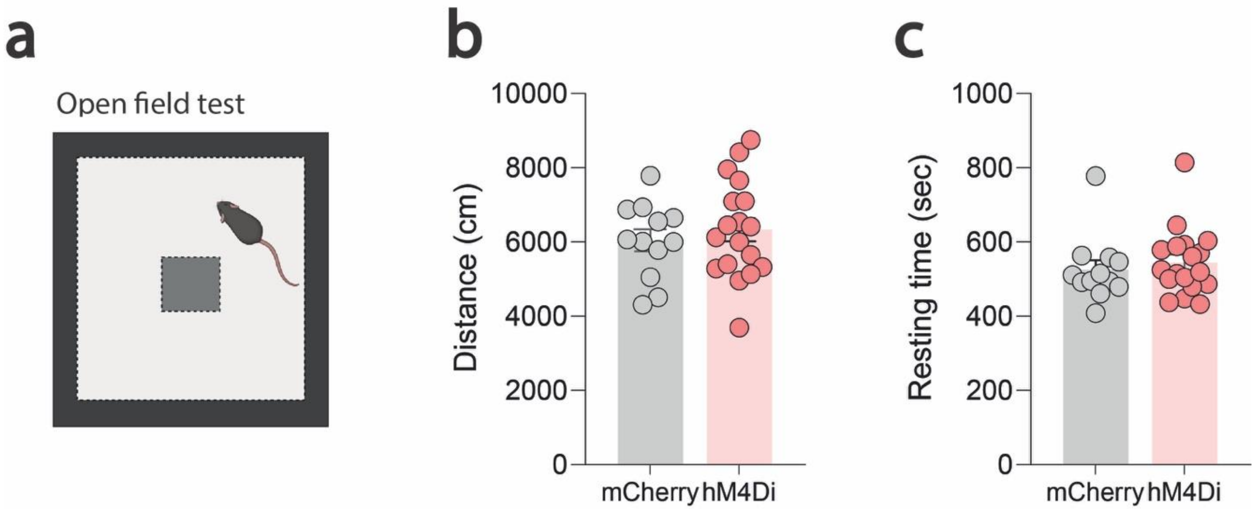

Fig. S13. mPFC→LHb neurons do not regulate exploratory or locomotor behaviors. (**a**) Graphical representation of the open-field apparatus. Mice expressing mCherry (*n*=12) or hM4Di (*n*=18) in cortico-habenular neurons were injected with CNO then allowed to freely explore the open-field apparatus for 30 min. (**b**) Distance travelled in the open field did not differ between groups. Unpaired two-tailed t-test: $t_{(28)}$=0.636, *p*=0.530. Data are expressed as mean (± SEM) total distance (cm) travelled. (**c**) Resting time in the open field did not differ between groups. Unpaired two-tailed t-test: $t_{(28)}$=0.577, *p*=0.569. Data are expressed as mean (± SEM) time (sec) spent at rest.

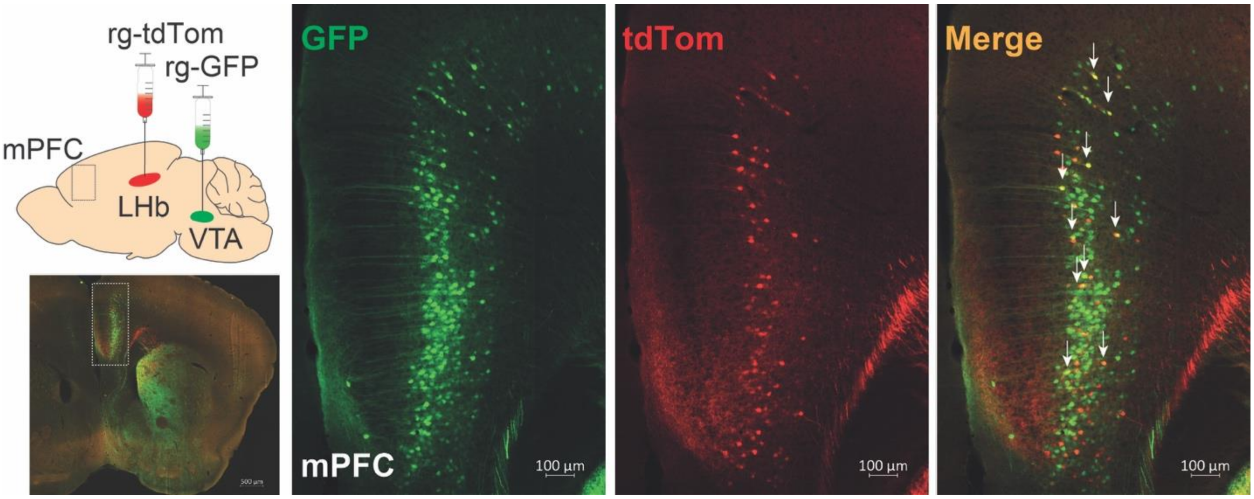

Fig. S14. Virus-based identification of mPFC$_{\rightarrow LHb}$^{VTA} neurons. (**a**) Graphical representation of the AAV-based retrograde labeling strategy used to visualize mPFC$_{\rightarrow LHb}$^{VTA} neurons (upper left panel). Infusion of rg-tdTom in the LHb and rg-GFP in the VTA resulted in the colocalization of tdTom and GFP in mPFC cells. A total of *n*=7 mice were imaged.

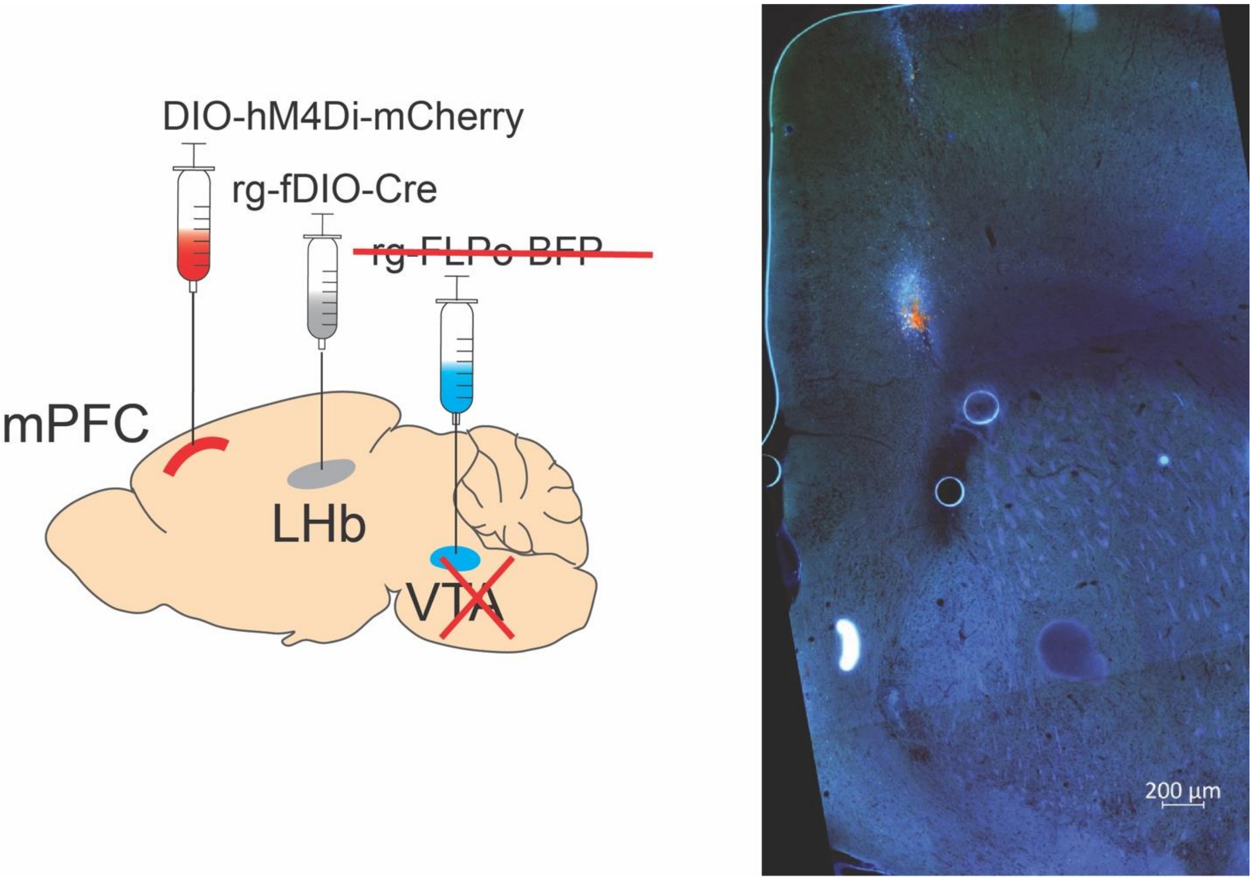

Fig. S15. Omission of viruses disrupts dual recombination strategy. Graphical representation of the virus strategy used to express hM4Di-mCherry (or only Cherry) in $mPFC_{\rightarrow LHb}^{VTA}$ neurons (left panel). In this case, the VTA injection of rg-FLPo was omitted (red lines). This resulted in the absence of any hM4Di-mCherry-expressing neurons in mPFC (right panel).

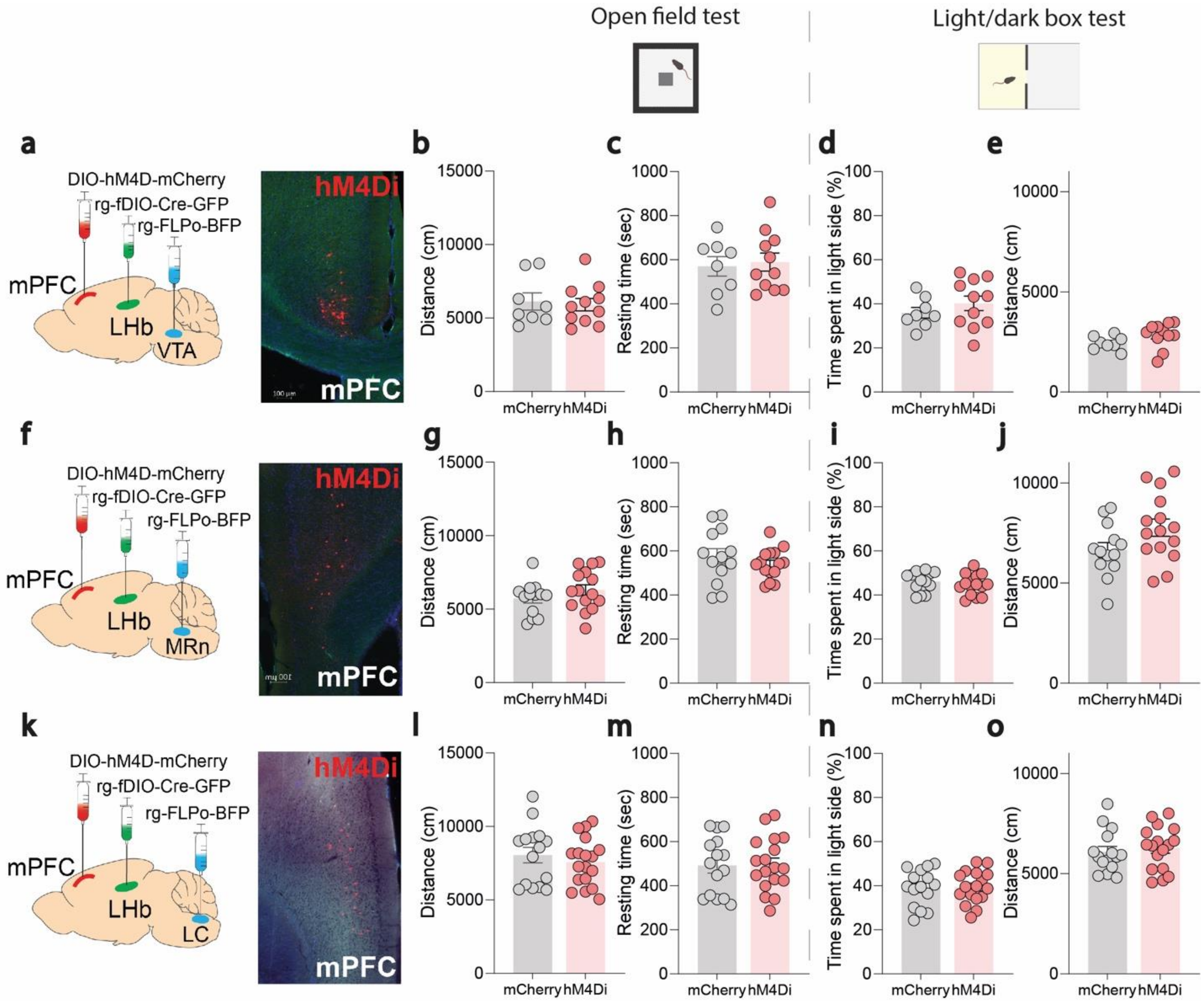

Fig. S16. Cortico-habenular neurons that project to VTA, MRn or LC do not regulate anxiety-related, exploratory and locomotor behaviors. (**a, f** and **k**) Graphical representations of the viral strategy used to selectively express mCherry or hM4Di-mChery in $mPFC_{\rightarrow LHb}^{VTA}$ (**a,** mCherry *n*=8, hM4Di *n*=11), $mPFC_{\rightarrow LHb}^{MRn}$ (**f,** mCherry *n*=13, hM4Di *n*=15) or $mPFC_{\rightarrow LHb}^{LC}$ (**k,** mCherry *n*=15, hM4Di *n*=18) neurons. Chemogenetic inhibition of cortico-habenular neurons that project to VTA (panels **b** and **c**), MRn (panels **g** and **h**) or LC (panels **l** and **m**) did not alter locomotor-related behaviors in an open field apparatus. Unpaired two-tailed t-tests for locomotor activity: $mPFC_{\rightarrow LHb}^{VTA}$: $t_{(17)}$=0.315, *p*=0.757; $mPFC_{\rightarrow LHb}^{MRn}$: $t_{(26)}$=1.195, *p*=0.243; $mPFC_{\rightarrow LHb}^{LC}$: $t_{(31)}$=0.746, *p*=0.460). Unpaired two-tailed t-tests for resting time: $mPFC_{\rightarrow LHb}^{VTA}$: $t_{(17)}$=0.312, *p*=0.759; $mPFC_{\rightarrow LHb}^{MRn}$: $t_{(26)}$=0.964, *p*=0.344; $mPFC_{\rightarrow LHb}^{LC}$: $t_{(31)}$=0.117, *p*=0.908. Chemogenetic inhibition of cortico-habenular neurons that project to VTA (panels **d** and **e**), MRn (panels **i** and **j**) or LC (panels **n** and **o**) did not alter anxiety- or exploratory-related behaviors in a light-dark box. Unpaired two-tailed t-tests for percentage of total time spent in the light side of the light-dark box: $mPFC_{\rightarrow LHb}^{VTA}$: $t_{(17)}$=0.971, *p*=0.345; $mPFC_{\rightarrow LHb}^{MRn}$: $t_{(26)}$=0.823, *p*=0.418; $mPFC_{\rightarrow LHb}^{LC}$: $t_{(30)}$=0.121, *p*=0.904. Unpaired two-tailed t-tests for distance travelled in the light-dark box: $mPFC_{\rightarrow LHb}^{VTA}$: $t_{(17)}$=1.619, *p*=0.124; $mPFC_{\rightarrow LHb}^{MRn}$: $t_{(26)}$=1.928, *p*=0.065; $mPFC_{\rightarrow LHb}^{LC}$: $t_{(30)}$=0.523, *p*=0.605.

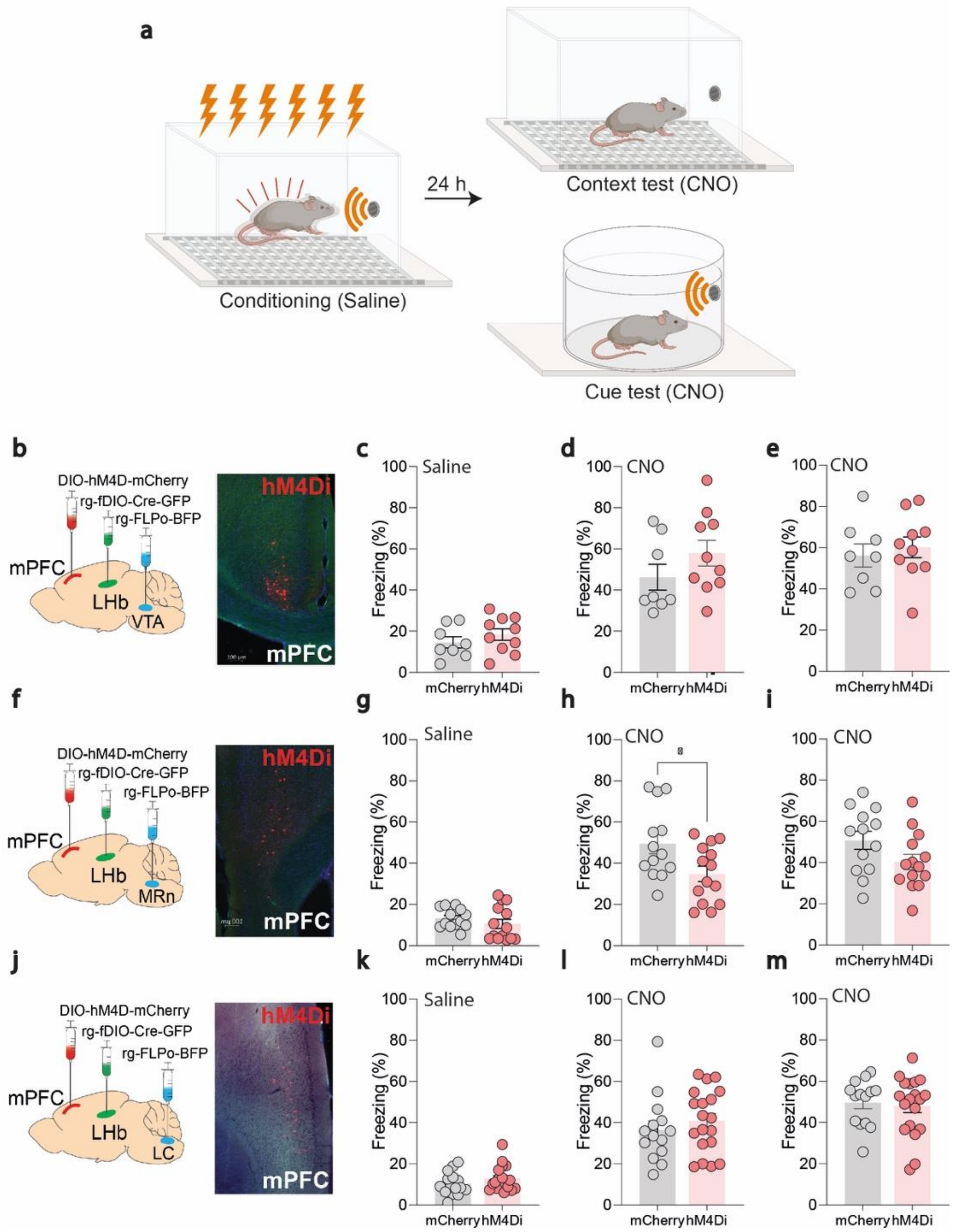

Fig. S17. Cortico-habenular neurons that project to MRn, but not to VTA or LC, regulate context-induced freezing. (a) Graphical representation of the fear conditioning procedure. Mice expressing mCherry or hM4Di in cortico-habenular neurons that project to VTA, MRn or LC were injected with saline then trained in the fear conditioning procedure during which noxious footshocks were delivered in a test apparatus in conjunction with a sound cue. 24 h later, all mice were injected with CNO and their context- and cue-induced freezing behavior was assessed. (**b, f** and **j**) Graphical representations of the viral strategy used to selectively express mCherry or hM4Di-mChery in cortico-habenular neurons that project to VTA (**b,** $mPFC_{\rightarrow LHb}^{VTA}$ neurons; mCherry *n*=8, hM4Di *n*=10), MRn (**f,** $mPFC_{\rightarrow LHb}^{MRn}$ neurons; mCherry *n*=13, hM4Di *n*=14 or LC (**j,** $mPFC_{\rightarrow LHb}^{LC}$ neurons; mCherry *n*=15, hM4Di *n*=18). (**c, g** and **k**) Freezing behavior during the conditioning session was similar between groups in all cases. Unpaired two-tailed t-tests for freezing between paired groups: $mPFC_{\rightarrow LHb}^{VTA}$: $t_{(16)}=0.951$, $p=0.359$; $mPFC_{\rightarrow LHb}^{MRn}$: $t_{(25)}=1.047$, $p=0.305$; $mPFC_{\rightarrow LHb}^{LC}$: $t_{(31)}=1.290$, $p=0.207$. (**d, h** and **l**) Context-induced conditioned freezing behavior was reduced by chemogenetic inhibition of cortical-habenular neurons that project to MRn (**h**), but not VTA (**d**) or LC (**l**). Unpaired two-tailed t-tests for freezing between paired groups: $mPFC_{\rightarrow LHb}^{MRn}$: $t_{(25)}=2.427$, $*p=0.023$; $mPFC_{\rightarrow LHb}^{VTA}$: $t_{(16)}=1.310$, $p=0.209$; $mPFC_{\rightarrow LHb}^{LC}$: $t_{(31)}=0.797$, $p=0.432$. (**d, h** and **l**) Cue-induced conditioned freezing behavior was unchanged by chemogenetic inhibition of cortical-habenular neurons that project to VTA (**e**), MRn (**i**) or LC (**m**). Unpaired two-tailed t-tests for freezing between paired groups: $mPFC_{\rightarrow LHb}^{VTA}$: $t_{(16)}=0.525$, $p=0.607$; $mPFC_{\rightarrow LHb}^{MRn}$: $t_{(25)}=1.837$, $p=0.078$; $mPFC_{\rightarrow LHb}^{LC}$: $t_{(31)}=0.346$, $p=0.732$.
